# Scalable and Degradable Dextrin-Based Elastomers for Wearable Touch Sensing

**DOI:** 10.1021/acsami.2c15634

**Published:** 2022-12-14

**Authors:** Xiaohong Lan, Wenjian Li, Chongnan Ye, Laura Boetje, Théophile Pelras, Fitrilia Silvianti, Qi Chen, Yutao Pei, Katja Loos

**Affiliations:** † Macromolecular Chemistry & New Polymeric Materials, Zernike Institute for Advanced Materials, University of Groningen, Nijenborgh 4, Groningen9747AG, The Netherlands; ‡ Advanced Production Engineering, Engineering and Technology Institute Groningen, University of Groningen, Nijenborgh 4, Groningen9747AG, The Netherlands

**Keywords:** dextrins, elastomers, bottlebrushes, lactones, sensors

## Abstract

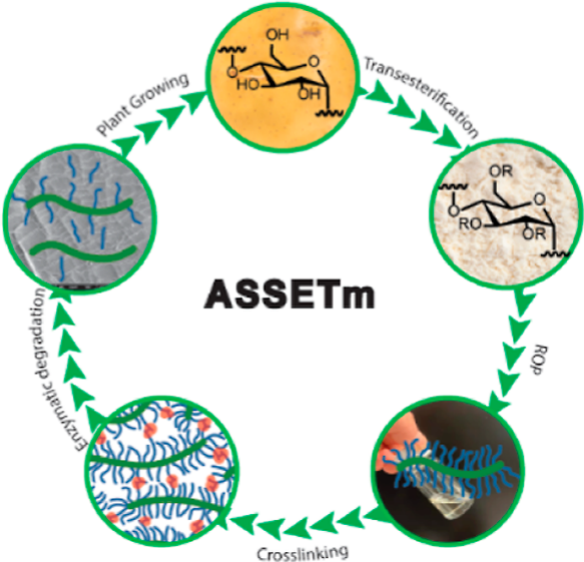

Elastomer-based wearables can improve people’s lives; however, frictional wear caused by manipulation may pose significant concerns regarding their durability and sustainability. To address the aforementioned issue, a new class of advanced scalable supersoft elastic transparent material (ASSETm) is reported, which offers a unique combination of scalability (20 g scale), stretchability (up to 235%), and enzymatic degradability (up to 65% in 30 days). The key feature of our design is to render native dextrin hydrophobic, which turns it into a macroinitiator for bulk ring-opening polymerization. Based on ASSETm, a self-powered touch sensor (ASSETm–TS) for touch sensing and non-contact approaching detection, possessing excellent electrical potential (up to 65 V) and rapid response time (60 ms), is fabricated. This work is a step toward developing sustainable soft electronic systems, and ASSETm’s tunability enables further improvement of electrical outputs, enhancing human-interactive applications.

## Introduction

Next generation of electronic devices, known as wearables, are quickly gaining interest in a variety of applications, such as on-skin wearable devices or biomedical implants,^1,2^ thanks to their adaptability and flexibility. These devices are currently built using elastomers [e.g., poly(dimethylsiloxane) (PDMS), elastomeric poly(d, l-lactide) (PLA) composites, polyurethane, polyacrylate, or hydrogels], profiting from their lightweight, stretchable, and inexpensive character. However, the practical use of those elastomer-based wearables is often hindered by their inherent shortcomings, potentially leading to serious safety and stability issues despite their promise to improve people’s lives.^3,4^ For instance, PLA composites—albeit being biobased—lack transparency and heat stability,^5,6^ hydrogels quickly dry out and crack,^7^ while polyurethane and polyacrylate elastomers may leach out unreacted and potentially toxic monomers.^8−12^ PDMS is widely used both in industry and research due to its affordable price and ease of use, but it is not degradable, limiting its use in wearables.^13^

In wearables, such as a triboelectric nanogenerator (TENG)-based touch sensor (TS), effective contact is typically required, which inevitably gives rise to frictional wear that can damage the sensor surfaces and their underlying structures, producing electronic waste (e-waste).^14^ To address these issues, degradable alternatives are studied. For instance poly[octamethylene maleate (anhydride) citrate] (POMAC)^15^ was previously used to fabricate a strain sensor to monitor the mechanical forces on tendons after surgical repair. In this system, however, the manipulation became challenging and fatigue life got limited because of the POMAC’s sticky gel nature. By using poly(1,8-octanediol-*co*-citrate-*co*-caprolactone),^16^ the sticky gel aspect was improved, but the gel contents showed that the majority of PCL is not in the crosslinked network, which may impart stability issues.

Bottlebrush polymers offer an opportunity into low modulus elastomers with high stability because of their densely dispersed dangling chains (full crosslinking is not necessary), showing promises for wearable applications. However, their synthetic strategies often require complex and costly procedures, impeding commercialization.^17,18^ Most importantly, most of these bottlebrush polymers are made of non-degradable acrylates, so clever macromolecular design is required to replace both backbone and side chains with appropriate bio-based materials.

Dextrin, a group of low-molecular-weight carbohydrates produced from the hydrolysis of starch and glycogen, is an ideal candidate for the bottlebrush backbone. Each repeating unit possesses three hydroxy groups that can be used as macroinitiators for the ring-opening polymerization (ROP) of cyclic esters, that is, growth of side chains via a grafting-from approach. However, the hydrophilic nature of native dextrin typically hampers its ability to control polymerization effectively.^19−21^ Partial chemical modification of dextrin to enable its solubility into lactone monomers for bulk polymerization may offer an opportunity for producing environmentally friendly wearable devices. Moreover, the bulky nature of the macroinitiator not only provides a steric barrier to prevent side reactions such as transesterification^22,23^ but also lowers the materials’ modulus due to steric repulsion between their side chains.^24^ Among cyclic esters, ε-caprolactone (ε-CL) is often a candidate of choice for the production of degradable plastics, benefiting from short reaction times, efficient catalytic systems, and the possibility to conduct polymerization in bulk. However, PCL is highly crystalline, so the introduction of a comonomer, such as δ-valerolactone (δ-VL), is regularly employed to inhibit PCL crystallization by impeding chain folding and alignment.^25,26^

**Scheme 1:**
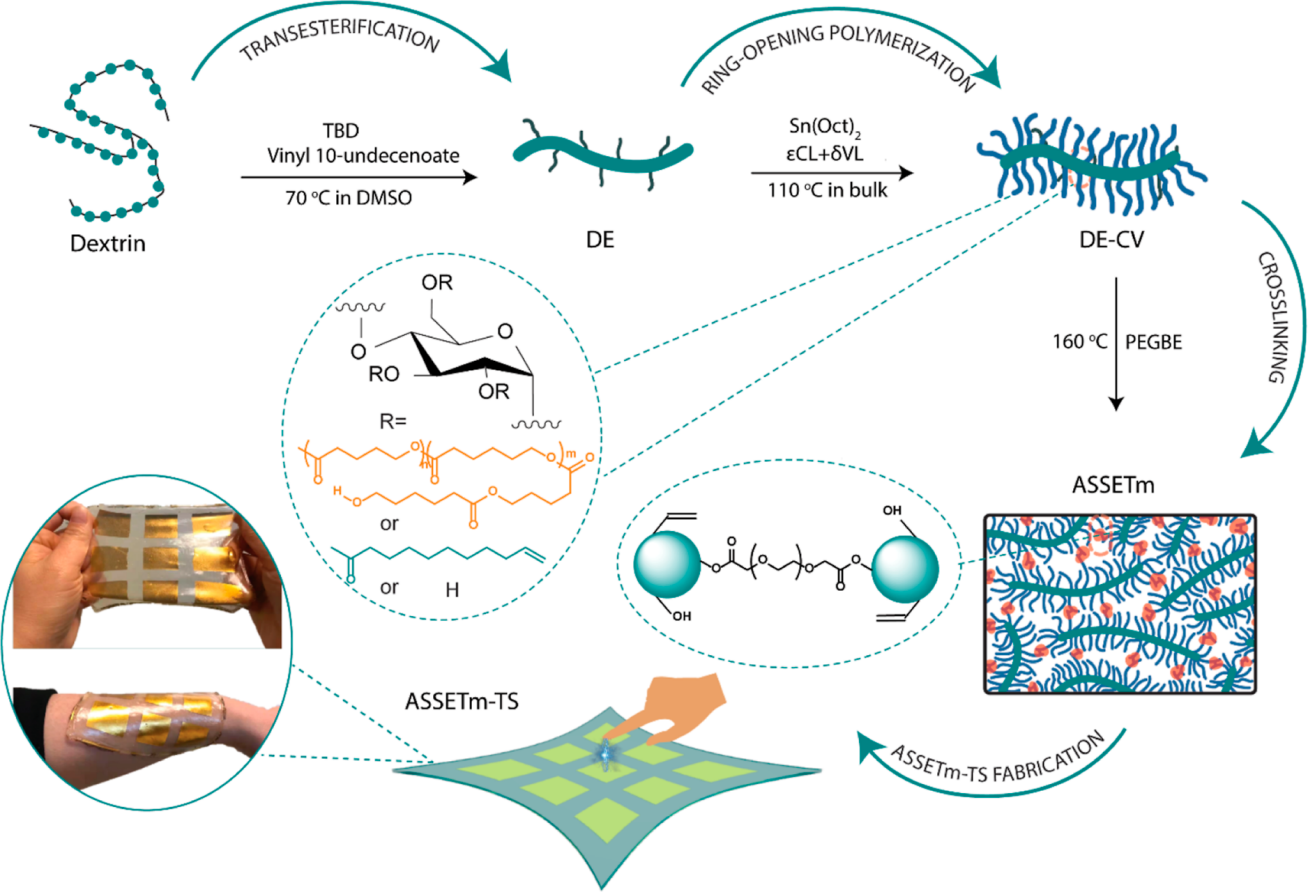
Schematic Illustration of the Preparation Process of Wearable ASSETm–TS

Herein, we have innovatively combined the fabrication of bottlebrush polymers from modified dextrin and lactones with the fabrication of soft elastomers for wearable electronics, as shown in Scheme 1. Making native dextrin hydrophobic is a crucial component of our strategy because it transforms it into a macroinitiator for bulk ROP. For the first time, an advanced scalable supersoft elastic transparent material (ASSETm) based on dextrin with good scalability and enzymatic degradability has been created. Following the successful elastomer synthesis, we have fabricated a sustainable self-powered ASSETm–TS for wearable touch sensing and non-contact approaching detection. The design flexibility of ASSETm facilitates the further improvement of electrical outputs, benefiting numerous human-machine interface applications and offering promising routes to enjoy a smart life while ensuring environmental sustainability.

## Results and Discussion

**Figure 1:**
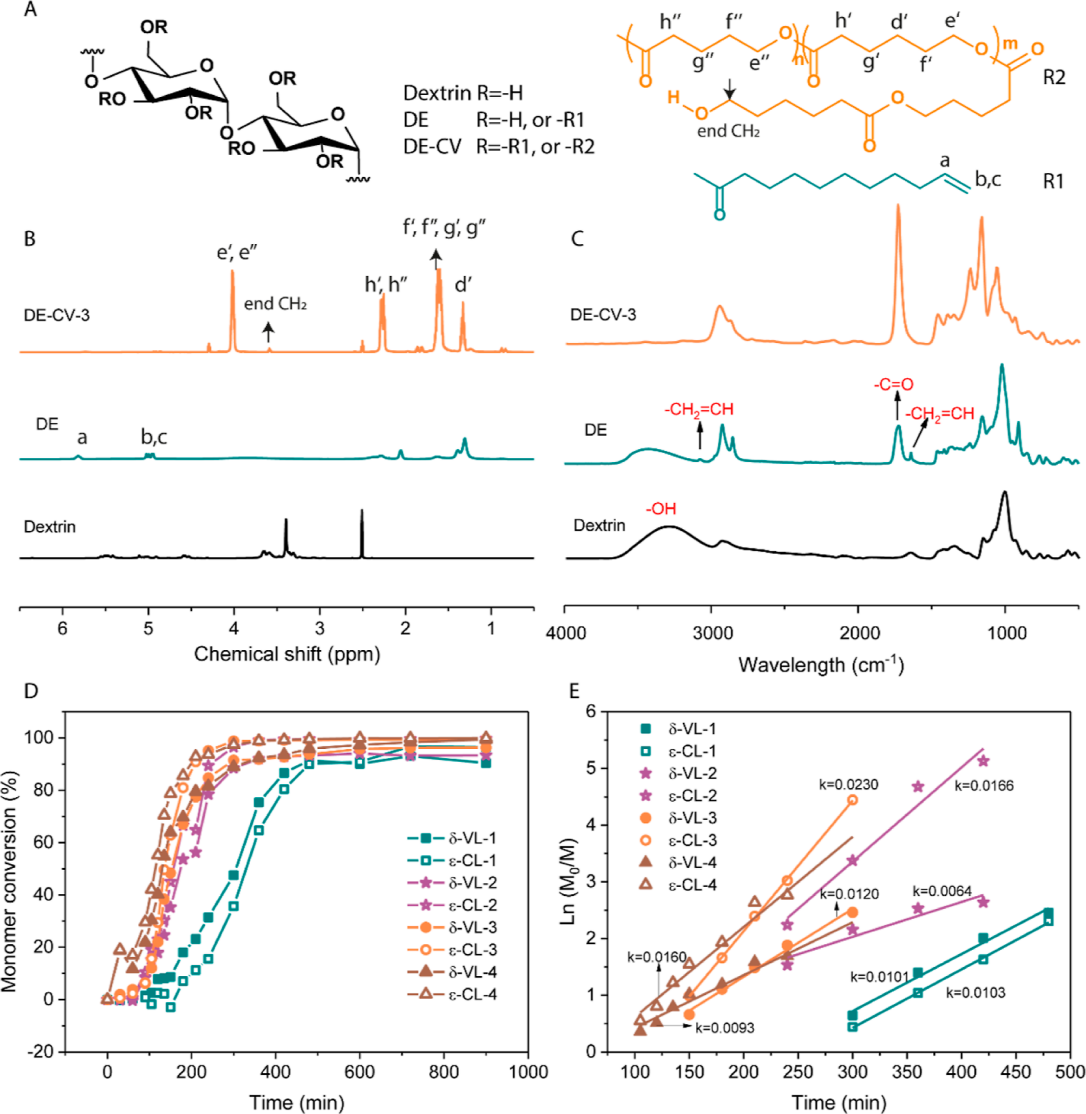
Characterization of dextrin and its derivatives. (A) Structure of dextrin and its derivatives. (B) ^1^H NMR spectra of the dextrin (in DMSO-*d*_6_), DE (in CDCl_3_), and DE–CV-3 (in CDCl_3_). (C) FTIR spectra of the dextrin, DE, and DE–CV-3. Kinetics of the ROP of ε-CL and δ-VL from the DE macroinitiator with varying *M*/*I* ratios evidenced by (D) conversion vs time plots and (E) logarithmic plot.

ASSETm was produced through a combination of (i) chemical modification of dextrin, (ii) ROP of lactones, and (iii) chemical cross-linking. Due to the insolubility of dextrin in the lactone monomers, we first performed an ester modification of native dextrin using 1,5,7-triazabicyclodec-5-ene (TBD) as a catalyst. Vinyl 10-undecenoate was chosen for its natural origin and the presence of a terminal vinyl group that facilitates the determination of the degree of substitution (DS). The structure of dextrin ester (DE) is depicted in Figure 1A. The vinyl signals of DE at 5.77 and 4.98 ppm confirmed the successful esterification and enabled the determination of DS (DS = 1.10) (Figure 1B). Further confirmation was obtained through Fourier transform infrared spectroscopy (FTIR, Figure 1C), with the appearance of the C=O stretch at 1735 cm^–1^ as well as the C=C stretches at 1600 and 3100 cm^–1^, alongside a decrease of the O–H stretch from dextrin at 3300 cm^–1^. Size exclusion chromatography (SEC, Figure S1) using DMSO as the eluent confirmed the preservation of the polymeric nature of the modified dextrin.

Next, the remaining hydroxy groups of DE were used as initiation sites for the stannous octoate [Sn(Oct)_2_]-catalyzed ROP of lactones. To synthesize a supersoft material with polyester side chains, ε-CL and δ-VL were chosen in virtue of their mutual interference on their crystallization profile.^25^ We produced a series of lactone-grafted DEs (DE–CV-*X*, *X* = 1, 2, 3, or 4) by varying the monomer to initiating site ratio (*M*/*I*, Table S1). Due to the complete depletion of lactones, the reaction was stopped after 7 h at 110 °C, and the polymer brushes were employed without further purification. ^1^H NMR analyses performed on the DE–CV polymer brushes feature the characteristic signals of PCL and PVL at 4.05, 2.30, 1.64, and 1.36 ppm (see Figures 1B and S2). Furthermore, the peak at around 3.60 ppm represents the terminal CH_2_ and was used to calculate the length of the brushes’ side chains (Figure S2).^27^

The growth of the PCL-*co*-PVL side chains was further validated by FTIR (Figure 1C), with a large increase of the C=O stretch at 1735 cm^–1^ originating from the multiple ester groups. Meanwhile, the O–H stretch at 3300 cm^–1^ is barely visible, superimposed by the numerous polylactone side chains. DMSO-based SEC (Figure S1) also confirmed the grafting of side chains from the DE macroinitiator, with a significant shift of the polymer peak toward lower retention times (i.e., high apparent molecular weights). The poor solubility of PCL-*co*-PVL in DMSO, however, does not permit the analysis of DE–CV-*X* with higher *M*/*I* ratios; therefore, chloroform-based SEC was used (Figure S3). The elugrams of the polymer brushes confirm the growth of the side chains, with the polymer signals shifting further toward lower retention times when higher *M*/*I* ratios were used (i.e., longer side chains and higher apparent molecular weights). Note that a secondary peak arises for *M*/*I* of 1:20 and above. This could originate from the multi-reaction mechanism behind the ROP (e.g., common coordination-insertion mechanism and activated monomer mechanism)^28^ and the dextrin’s dispersity. Nonetheless, to avoid selective fractionation in organic solvents, we retained these lower molecular weight polymer species for the later cross-linking process and the fabrication of ASSETm–TS.

We further investigated the kinetics of the ROP of lactone from the macroinitiator DE using ^1^H NMR. Monomer conversion versus reaction time plots (Figure 1D) reveal relatively quick monomer consumption, with all the reactions running into a plateau after 7 h. Logarithmic plots of the relative monomer concentration versus polymerization time (Figure 1E) further evidenced the presence of an induction time, inversely proportional to the *M*/*I* ratio. This likely originates from the higher viscosity of the reaction mixture with lower *M*/*I* ratios that hinders structural rearrangements of the macroinitiator from exposing active sites.^29^ Once the induction time passed, the rate of polymerization follows a first-order dependence on monomer concentration, with a linear relationship between ln (*M*_0_/*M*) and time. The apparent polymerization rate constant *n* is equal to the estimated slope *k* of the linear component of the curve. Surprisingly, in DE–CV-2, DE–CV-3, and DE–CV-4 samples, the polymerization rate of ε-CL was higher than that of δ-VL, the latter being reported as a faster-propagating monomer due to its high ring strain.^30^ This was attributed to the catalyst-initiator complex’s varied incorporation preferences for monomers. This preference for monomers can be tuned by either employing a different catalyst or initiator, as their chemical natures have a major impact on the equilibrium between the free and protonated catalyst-initiator complex.^31,32^

Then, DE–CV-*X* was crosslinked by reacting the terminal hydroxy groups of PCL-*co*-PVL side chains with the carboxylic groups of poly(ethylene glycol) bis (carboxymethyl) ether (PEGBE), catalyzed by Sn(Oct)_2_ that was left in the previous ROP step. DE–CV-*X* and PEGBE were dissolved in acetone, the solution was poured into a teflon plate, and the solvent was left to evaporate before exposing the films to 160 °C for 5 h, yielding our ASSETm-*X* (*X* = 1, 2, 3, and 4, e.g., ASSETm-1 refers to the elastomer made of DE–CV-1). For all film castings, we used 0.25 equiv of the carboxylic group per hydroxy group to ensure complete crosslinking, while preserving sufficient dangling polylactone chains. An appropriate proportion of dangling chains can significantly reduce material’s modulus by preventing the formation of entanglements,^33^ increasing the flowability of the network. All ASSETm displayed a rubbery behavior with no visual sign of flowing. Gel fraction measurements (Figure S4) were performed to monitor the potential leaching of the non-crosslinked material. As the *M*/*I* ratio increases, there is a marginal decrease in the gel fraction (determined by eq 2). Nonetheless, ASSETm-4 still possesses a high gel fraction of 86%, which indicates that the vast majority of brushes have been linked into the network. Yet, our ASSETm remains more stable than commercially available EcoFlex (gel fraction of 60%).^34^

**Figure 2:**
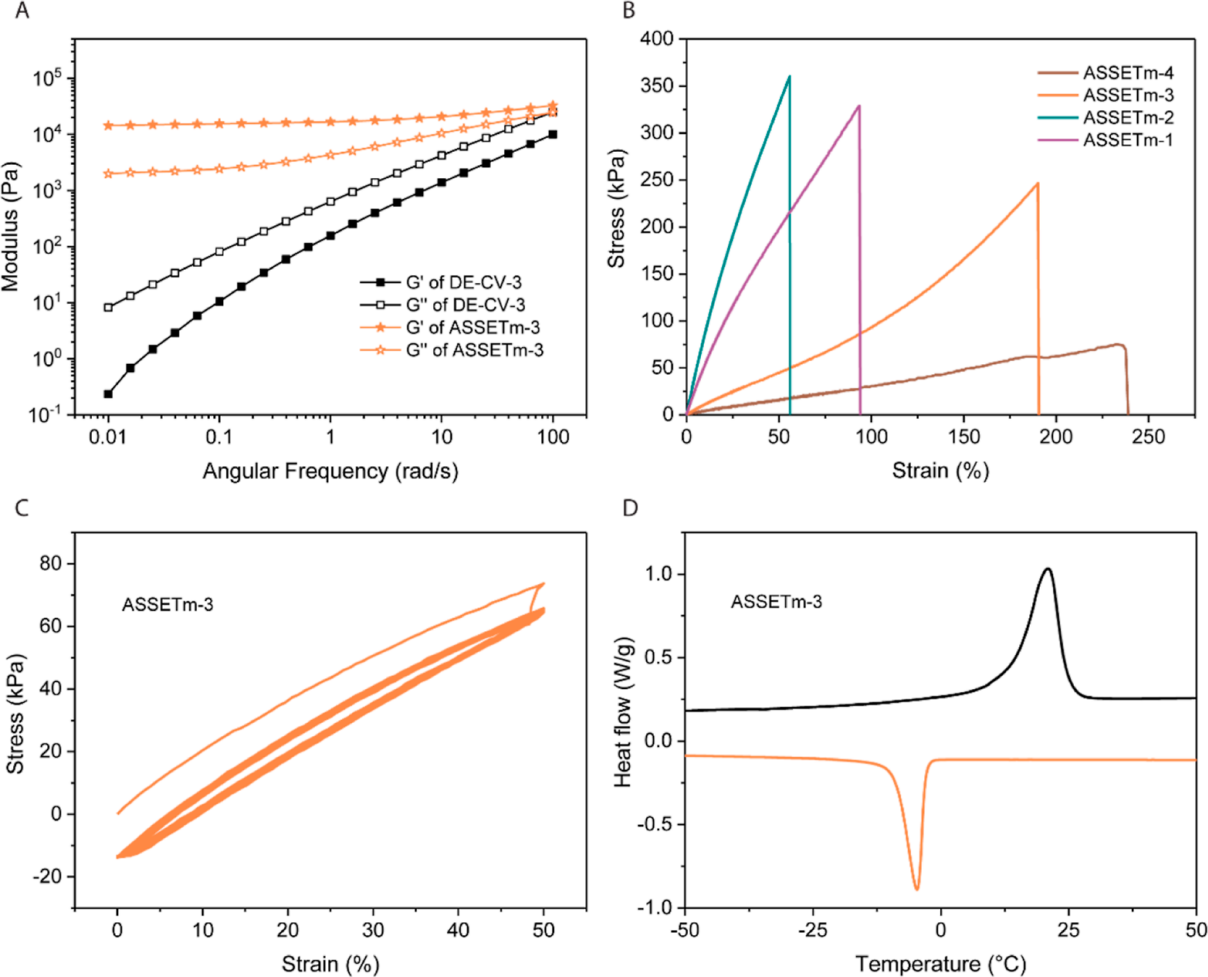
Fabrication and characterization of ASSETm. (A) Frequency dependence of the storage and loss modulus of the DE–CV-3 and ASSETm-3. (B) Uniaxial tensile test of the ASSETm. (C) Cycling test of the ASSETm-3. (D) DSC profile of the ASSETm-3.

To further confirm proper network integrity, oscillatory rheology was used to monitor the viscoelasticity of the materials before and after crosslinking. All ASSETm showed a prominent plateau in *G*′ with nearly frequency-independent shear storage moduli, resembling a perfect rubber (Figures 2A and S5), while the modulus of uncured DE–CV-*X* largely increased with the increase in frequency. Additionally, ASSETm-1 displayed a higher viscoelastic behavior, with both storage and loss moduli 1 order of magnitude above the rest of the materials. Furthermore, tensile tests were performed on the various ASSETm, which yielded the characteristic stress–strain curves of elastomeric materials (Figure 2B). Samples produced with a higher *M*/*I* ratio evidenced higher elongation at break (60% for ASSETm-1 vs 230% for ASSETm-4), while the Young’s modulus dropped from 9.04 to 0.28 MPa (Table S2). It is worth mentioning that all the ASSETm possess a Young’s modulus within the range of that of muscle tissue (10–500 kPa)^35^ and is significantly lower than that of the silicon-based elastomer Sylgard 184 (Figure S6).^33^ Overall, the mechanical properties of our elastomers are similar to that of PCL crosslinked by polyhedral oligomeric silsesquioxane,^36^ yet they were produced following a greener approach. Unlike 100% natural polymers such as gelatin^37^ and cellulose,^38^ the mechanical properties of our ASSETm can easily be tuned through a simple change of the *M*/*I* ratio (i.e., shorter or longer side chains) and benefit from water and organic solvent resistance. To verify their mechanical stability, the elastomers were subjected to repeated force loading–unloading (Figure 2C), where the properties remained unaltered after the first cycles. This contrasts with linear PCL, which exhibits severe hysteresis in stretching–relaxation cycles, resulting in mechanical loss under cyclic loading.^39^

Next, we investigated the thermal properties of our ASSETm. Thermogravimetric analysis (TGA, Figure S7) evidenced an earlier degradation profile for the DE compared to the pristine dextrin, which originates from the introduction of fatty acid. However, once crosslinked, the thermal stability of the elastomer significantly improves, thanks to its crosslinked nature. The ASSETm starts to degrade at ∼260 °C with a maximal burn rate at ∼300 °C (Table S3), which is beyond the requirements for wearables. Differential scanning calorimetry (DSC, Figures 2D and S8) analyses of the elastomers show, that all ASSETm possess a melting temperature *T*_m_, which increases from 6.2 °C for ASSETm-1 to 21.0 °C for ASSETm-3 and ASSETm-4, and correlates to the increased length of the PCL-*co*-PVL side chains. It is important to note that a cold crystallization peak for ASSETm-1 was observed (*T*_cc_ = −16.4 °C), ascribing to an exothermic crystallization process occurring upon heating. This does not occur for ASSETm-2 and above, possibly due to the non-formation of stable nuclei.

**Figure 3:**
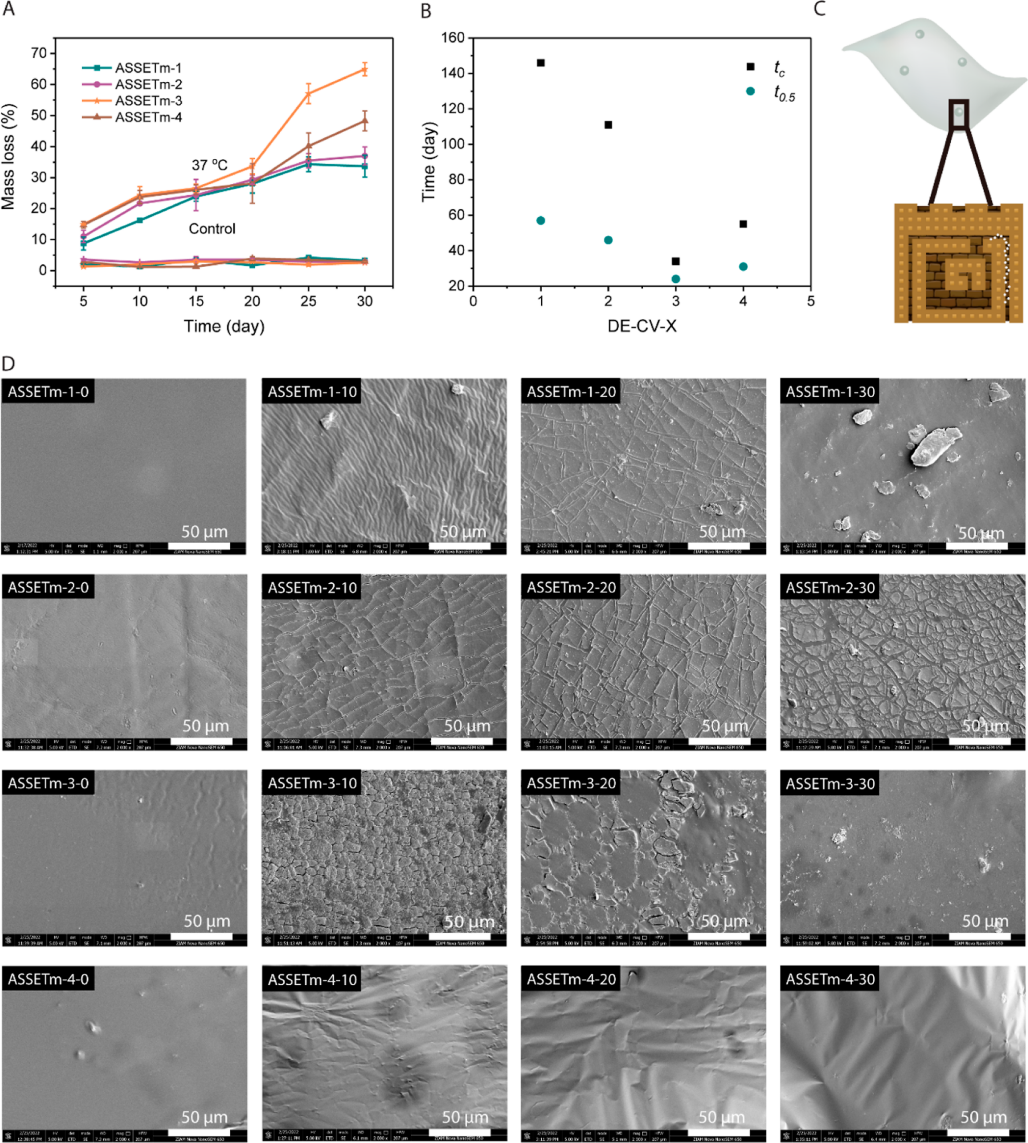
Enzymatic degradability of ASSETm. (A) ASSETm’s mass loss with enzymatic degradation at 37 °C. (B) The predicted half degradation time (*t*_0.5_) and complete degradation time (*t*_c_) of the ASSETm. (C) A schematic illustrating how lipase prefers to degrade PVL and create channels (white dots represent lipase). (D) Surface morphology changes of the ASSETm with degradation.

ASSETm is a bottlebrush-based thermoset having dextrin as its backbone and PCL-*co*-PVL as its grafting side chains. Dextrins, which are derived from starch, have been shown to be biodegradable.^32^ Although PCL, a linear aliphatic polyester, is supposed to be degradable; however, its crystalline structure severely restricts this ability. The degradability of ASSETm is due to the grafted PCL-*co*-PVL, which has an amorphous structure and high susceptibility to enzymatic degradation.^40^ The progress of the degradation might be delayed by the crosslinks, which give the polymers more dimensional stability. However, inherently low modulus and large dangling chains would still play an essential role in degradability.^41^ Degradability tests were performed in vitro at 37 and 25 °C in PBS buffer containing 5 U mL^–1^ lipases. Following 30 days of incubation, all samples maintained their original shape, while exhibiting a gradual mass loss (determined by eq 3) during incubation, as shown in Figure 3A. After incubation at 37 °C for 30 days, ASSETm-3 shows the highest mass loss of 65%, while ASSETm-4 shows the second highest mass loss of 48%. The half degradation time *t*_0.5_ of ASSETm, as predicted from the mass loss curve, is shown in Figure 3B. With the increase in *M*/*I*, the *t*_0.5_ of the ASSETm decreased first and then increased. Samples with low *M*/*I* tend to have more rigid mechanical features due to less flexible side chains, hence hindering the accessibility to enzymes resulting in slower degradation rates. However, compared to ASSETm-3, ASSETm-4 films tend to aggregate together during incubation, therefore, reducing their degradability. In the meantime, the complete degradation time *t*_c_ of the ASSETm was also predicted from the mass loss, as shown in Figure 3B. Because the degradation follows a surface degradation mechanism (Figure 3C),^42^ the practical *t*_c_ should be much lower than the predicted ones. To make the degradation process more visible, the surface morphology of sample films was monitored by SEM throughout the whole incubation duration, as shown in Figure 3D. Before degradation, all of the samples possess a smooth surface, but after 10 days, wrinkles, fragments, and channels were observed on the surface. As the channels grew larger, the surface layer was eroded and exfoliated into brick-like shards, and a fresh layer was produced in the end. The channels observed could be attributed to the two polymers subjected to enzymes having different degradation preferences. However, we could not capture the fragmentation process with ASSETm-4; the surface remained rather smooth throughout the degradation period, with only some wrinkles. This could be because of its lengthy side chains, which facilitate its surface self-healing during and after degradation. These results show that ASSETm are enzymatically degradable elastomers, and the degradation characteristics of ASSETm are expected to fall within the degradation scale required for most applications, such as soft electronics. Higher incubation temperatures boost enzyme activity, resulting in substantially shorter periods of degradation (Figures 3A and S9), and the temperature utilized in our research (37 and 25 °C) are instructional for various environmental degradation processes, including biological degradation and soil composting.

PDMS-based elastomers such as Sylgard 184 are widely used in wearables because of their stretchability and ease of production. In terms of softness, stretchability, and degradability, the proposed ASSETm in our study outperforms Sylgard 184. Therefore, ASSETm is an excellent candidate for wearable applications, particularly for TENG-based TS where frictional wear is unavoidable, resulting in a significant amount of e-waste. Based on ASSETm, a single-electrode device was developed for touch sensing. The ASSETm–TS consists of three layers: two ASSETm layers with a gold electrode sandwiched in the middle, as illustrated in Figure S10. The working mechanism of the ASSETm–TS is based on triboelectrification and the electrostatic induction effect (Figure S11). As a result of triboelectrification, an equal amount of positive and negative charges are generated at the surface of the ASSETm–TS and the pressing object [here, a polytetrafluoroethylene (PTFE) film], respectively, when an object touches the device (state I). When the object separates from the device, the unbalanced surface charges on the ASSETm–TS drive electrons to flow from the ground to the gold electrode due to electrostatic induction. Therefore, an instantaneous electrical current (state II) is generated until the triboelectric charges on the device are thoroughly screened by induced charges (state III). Similarly, a reverse electron flow and current will occur when the object approaches the device (state IV).

**Figure 4:**
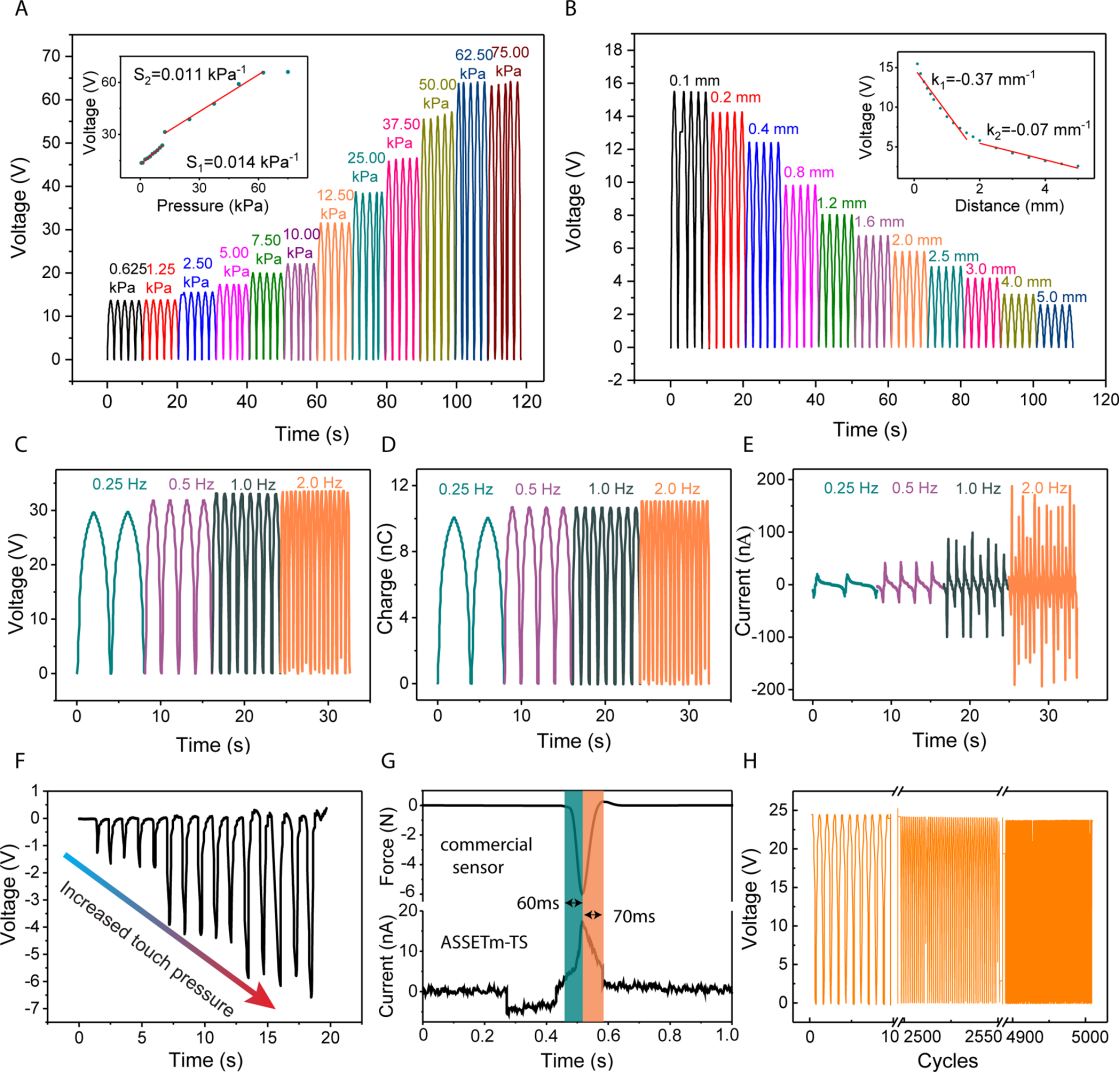
Self-powered ASSETm–TS for touch and non-contact motion detection. Voltage changes of the ASSETm–TS at different (A) pressures and (B) distances. (C) Voltage, (D) charge, and (E) current changes of the ASSETm–TS at different stimulation frequencies. (F) Voltage responses of the ASSETm–TS by pressing one pixel with a finger with the increase in pressures. (G) Response and relaxation time of the ASSETm–TS and a commercial sensor. (H) Stability test of the ASSETm–TS over ∼5000 cycles.

To quantitatively assess the electrical output performance of the ASSETm–TS, the open-circuit voltage, short-circuit current, and short-circuit transferred charges were investigated under different pressures, as displayed in Figures 4A and S12. Because of the increased effective contact area when the object touches the ASSETm–TS, the voltage output gradually increased before saturation (65 V) at a pressure of 62.5 kPa. The sensitivity of ASSETm–TS, *S*, as a function of loading pressure is calculated according to eq 1
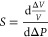
1where Δ*V* is the relative voltage changes, *V* is the saturated voltage, and Δ*P* is the relative pressure changes. As depicted in Figure 4A, the sensitivity in the low-pressure range (0.625–11.25 kPa) is 0.014 kPa^–1^, whereas the sensitivity in the high-pressure range (12.5–62.5 kPa) is 0.011 kPa^–1^, respectively. It is worth noting that the ASSETm–TS produces a high voltage output of 16 V even at a low pressure of 0.625 kPa, indicating enormous potential for low-pressure and even non-contact approach sensing. In advanced intelligent sensing, it is necessary not only to know the pressure that a pressing object exerts on the sensor but also to predict the exact position during the approaching process. Therefore, the non-contact approach sensing performance was examined by varying the distances between the object and the ASSETm–TS. Figure 4B demonstrates an increase of the output voltage from 2.6 to 15.5 V when the object gradually approached the ASSETm–TS by stopping at a distance of 5 to 0.1 mm. The sensitivity of the non-contact approach sensing in ASSETm–TS is 0.37 mm^–1^ for small distance ranges (0.1–1.5 mm) and 0.07 mm^–1^ for large distance ranges (1.5–5.0 mm). Figure 4C–E shows the electrical output of the ASSETm–TS under various stimulation frequencies. The current increased linearly from 21 to 172 nA as the frequency increased from 0.25 to 2 Hz, while the voltage and charge remained relatively constant. To demonstrate the capabilities of the wearable sensor system for real-time measurement, the ASSETm–TS was worn onto the arm and its electrical outputs to a fingertip touch with the increase in pressure were spontaneously recorded, which is shown in Figure 4F. With an increase in the fingertip pressure, the output voltage of the ASSETm–TS increased accordingly, demonstrating its good applicability as a wearable electronic skin and tactile sensor. Figure 4G compares one cycle of ASSETm–TS’s response with that of a commercial sensor (a pressure sensor in Instron 5565). The response time of ASSETm–TS, defined as the time needed for the output to increase to the full scale under pressure,^43^ is 60 ms, comparable to that of the commercial pressure sensor. The relaxation time, defined as the time it takes for the sensor to recover to its initial value before applying pressure, is 70 ms, suggesting that the ASSETm–TS has a fast response and no-delay recovery characteristics. The cyclic stability of our sensor over 5000 cycles was evaluated at a pressure of 12.5 kPa and a frequency of 1 Hz, and the results are presented in Figure 4H. There was only a trivial reduction in output, which can be attributed to the gradual compression of the double-sided foam tape used to fix the device to the testing machine. As such, the ASSETm–TS has an excellent performance in tracking the pressure, frequency, and location, demonstrating its immense prospects in a wide variety of applications for robotics, human-machine interaction, and security systems.

## Conclusions

In this study, we synthesized a novel dextrin-based elastomer ASSETm for the fabrication of TS. The whole processing does not require the use of toxic solvents, and all of the reactants are bio-based, making this a green polymer from head to toe. Modulating the *M*/*I* ratios, we can easily adjust the properties of our materials, including molecular weight, mechanical strength, and degradation rate. Compared to existing degradable elastomers, ASSETm is less expensive, easier to scale up and produced in a greener fashion, highlighting the ecofriendly possibilities of flexible electronic devices and pointing out design routes beyond silicone and polyurethane elastomers. Based on ASSETm, a self-powered touch-sensing device with a fast response time of 60 ms comparable to a commercial pressure sensor was fabricated. To effectively integrate ASSETm into wearables’ applications in the future, ongoing studies into its 3D printability and self-healing capability are currently conducted.

## Experimental Section

### Materials

Dextrin D4657 and vinyl-10-undecenoate (>92%) were purchased from TCI, ε-caprolactone (ε-CL, 97%), δ-valerolactone (δ-VL, technical grade), PEGBE (*M*_w_ = 600), TBD (98%), lipase from *Aspergillus oryzae* (L4277), phosphate-buffered saline (PBS, pH 7.4), and stannous octoate [Sn(Oct)_2_, 92.5–100%] were purchased from Sigma-Aldrich. All reagents were used as received, except [ε-CL, δ-VL, and Sn(Oct)_2_], which were dried over MgSO_4_ for 30 min before use. All other solvents were from Avantor and were of analytical grade.

### Instrumentation

NMR spectra were recorded at 25 °C on a Bruker Advance NMR spectrometer operating at 600 MHz. The following abbreviations were used to explain the multiplicities: s = singlet, d = doublet, t = triplet, q = quartet, m = multiplet, and br = broad. Molecular weights of polymers were determined by size extrusion chromatography (SEC) equipped with a triple detector, consisting of a Malvern Dual detector and a Schambeck RI2012 refractive index detector, as well as two PLgel (both 5 μm 30 cm) from Agilent Technologies at 35 °C. ATR–FTIR spectra were recorded on a Bruker Vertex 70 infrared spectrometer using a diamond ATR probe. DSC thermograms were recorded on a TA instruments Q1000 under nitrogen at heating and cooling rates of 10 °C min^–1^. A TA-instrumental D2500 under a nitrogen atmosphere was used for the TGA operating at 10 °C min^–1^. Uniaxial tensile and cyclic strain tests were performed on an Instron 5565 with a force of 100 N. The frequency sweep experiments were carried out on an Anton-Paar Physica MCR 302e rheometer with a plate–plate geometry of 25 mm using a strain of 1%. The surface morphology was examined by SEM (FEI Nova NanoSEM 650) operating at an accelerating voltage of 5 kV. Prior to imaging, the specimens were coated with 10 nm Au using a Cressington Sputter Coater 208HR. The tactile sensor measurement was conducted using a universal material test system (MTS 810), and a pressure sensor (Chino Sensor, ZNLBM-5KG) was used to measure the force. The voltage, charge, and current were measured by an electrometer (Keithley 6514).

### Synthesis of 10-Undecenoate DE

A specific amount of dextrin [2 g, 12.34 mmol anhydroglucose unit (AGU)] was dissolved in DMSO (20 mL) and heated for 30 min at 70 °C in a round-bottom flask. Once dissolved, vinyl-10-undecenoate (0.28 g, 1.1 equiv AGU) and TBD (0.086 g, 0.05 equiv AGU) were added to the dextrin solution. The reaction was stirred for 5 h at 70 °C. The light yellow solution was added dropwise to acetonitrile to precipitate the products. The purification process was repeated twice and the collected DE was vacuum-dried for further use.

Yield: 70%. ^1^H NMR (600 MHz, CDCl_3_, δ): 5.77 (br, 1H, −CH=), 5.45 (m, 2OH), 5.10 (br, 1H, −CH−), 4.98 (q, 2H, =CH_2_), 4.58 (m, 1OH), 3.74–3.24 (br, 6H), 2.31(br, 2H, −CH_2_−), 1.99 (s, 2H, −CH_2_−), 1.58 (s, 2H, −CH_2_−), 1.10–1.40 (d, 10H, −(CH_2_)_5_−).

### Synthesis of Bottlebrush Prepolymers (DE–CV-*X*)

To a 20 mL vial with a stir bar, a certain amount of DE (0.342 g, 2 mmol hydroxy group) as the initiator was introduced first and a mixture of ε-CL and δ-VL (with ε-CL/δ-VL = 1:1) as monomers was added later. The mixture was stirred at room temperature for 1 h to achieve complete dissolution, and then, dried Sn(Oct)_2_ was added before sealing the reaction vessel. The mixture was then stirred in a pre-heated oil bath at 110 °C. The conversion of monomer was evaluated by ^1^H NMR by removing 0.1 mL aliquots from the system at appropriate time intervals. Once the desired conversion was achieved, the vessel was removed from the oil bath and cooled over an ice bath. The concentrated and viscous products DE–CV-*X* were used after.

Yield: 99%. ^1^H NMR (600 MHz, CDCl_3_, δ): 5.77 (br, 1H, −CH=), 4.98 (q, 2H, =CH_2_), 4.05 (m, *n* × 2H, *n*-CH_2_–OOC−), 2.30 (m, *n* × 2H, *n*-COO–CH_2_−), 1.64 (m, *n* × 2 × 2H, *n*-CH_2_–CH_2_−), 1.36 (m, 0.5*n* × 2H, 0.5*n*-CH_2_−). The *n* denotes the molecular ratios of lactone monomers to hydroxy groups.

### Synthesis of ASSETm

The ASSETm were formed using solvent-casting and curing techniques. As an example, the synthesis of ASSETm-3 was described as follows: first, DE–CV-3 (4 g) was dissolved in acetone and vigorously stirred for several minutes. Then, the diluted PEGBE (60 mg) in acetone was added and the mixture was vigorously stirred. The well-mixed solution was degassed for 5 min before being poured onto a completely flat and well-polished teflon mold. After the solvent was evaporated, the material was heated for 8 h at 160 °C. All of the samples were cast to a thickness of approximately 0.25 mm. The sample names are codified according to *M*/*I*, ASSETm-1, ASSETm-2, ASSETm-3, and ASSETm-4, respectively.

### Gel Fraction Measurement

The ASSETm were analyzed by swelling experiments in acetone for 24 h at an ambient temperature, with another 24 h for solvent evaporation. This process was repeated twice to verify that all linear polymers were removed. The gelation degree was calculated by dividing the dry weight following extraction *m*_a_ by that of original sample *m*_b_, as shown in eq 2
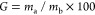
2

### Enzymatic Degradation

The enzymatic degradation behavior of ASSETm films (size 20 × 10 × 0.25 mm) was performed in a phosphate buffer solution (PBS) containing lipases (5 U mL^–1^) for 30 days at both 37 and 25 °C.^44^ At regular time intervals, the polymer specimens were removed from the degradation media and then washed with distilled water before being vacuum-dried at 45 °C to a constant weight. As a control, the hydrolytic degradation of the polymers without enzymes was also studied. Mass loss percentage following enzymatic degradation of the ASSETm can be calculated from the following equation
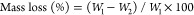
3where *W*_1_ and *W*_2_ denote the initial weight and dry weight of the sample film after degradation, respectively.

### Fabrication of the ASSETm-TS

The fabrication procedure of ASSETm–TS is described as follows. The first layer was produced by casting a mixture of DE–CV-3 and crosslinker onto the teflon mold and curing it at 160 °C for 5 h. The surface of ASSETm-3 was then coated with gold nanoparticles using a mask (Temescal FC 2000), forming a 100 nm thick gold electrode. The gold wire was then attached to the electrodes for electrical connection. The third layer was fabricated in the same manner as the first layer. The size of the ASSETm–TS is 10 × 10 cm^2^ with nine arrays, and the thickness of the entire ASSETm–TS is 0.5 mm.

## Supporting Information Available

NMR spectra, SEC elugrams, DSC analyses, TGA, rheology, tensile test, degradability, and electronical output data (PDF)
