# Supplementary material for: Scalable and Degradable Dextrin-Based Elastomers for Wearable Touch Sensing
Source: ACS Appl Mater Interfaces. 2022 Dec 14;15(3):4398–407. doi: 10.1021/acsami.2c15634 (PMC9880951; doi:10.1021/acsami.2c15634)
Supplement: Supplementary file 1 — am2c15634_si_001.pdf [file am2c15634_si_001.pdf]

SUPPORTING INFORMATION

Scalable and degradable dextrin-based elastomers for wearable touch sensing

Xiaohong Lan,[1‡] Wenjian Li,[2‡] Chongnan Ye,[1‡] Laura Boetje,[1] Théophile Pelras,[1] Fitrilia Silvianti,[1] Qi Chen,[1] Yutao Pei,[2] and Katja Loos[1]*

[1] Macromolecular Chemistry & New Polymeric Materials, Zernike Institute for Advanced Materials, University of Groningen, Nijenborgh 4, 9747AG, Groningen, The Netherlands

[2] Advanced Production Engineering, Engineering and Technology Institute Groningen, University of Groningen, Nijenborgh 4, 9747AG, Groningen, The Netherlands

[*]Corresponding author email

Katja Loos: k.u.loos@rug.nl

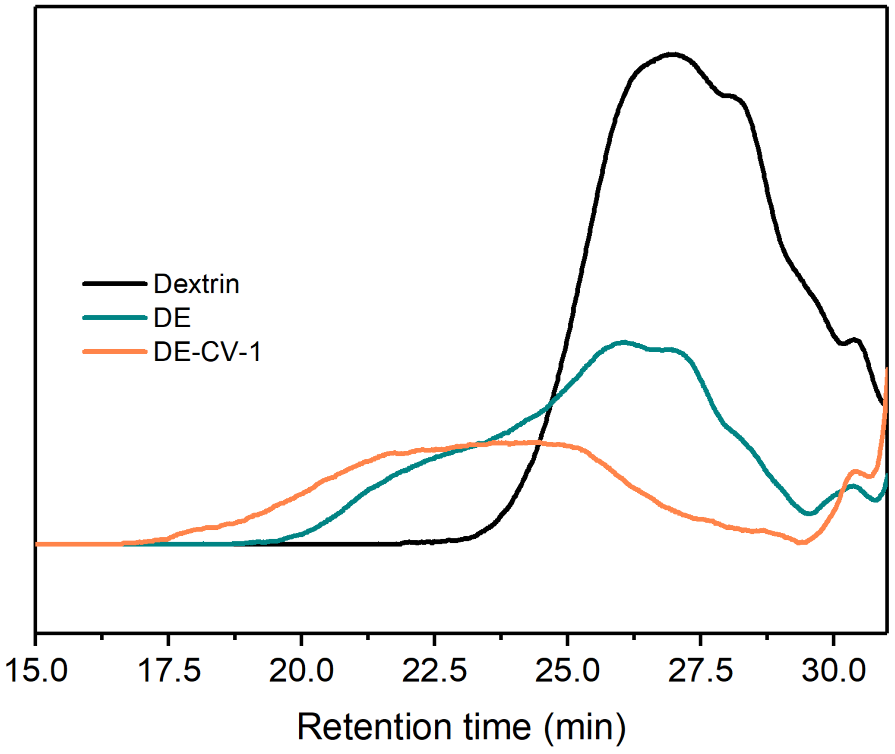

Figure S1. SEC elugrams of the native dextrin, DE, and DE-CV-1, measured at 80 °C in DMSO with 0.01 M LiBr.

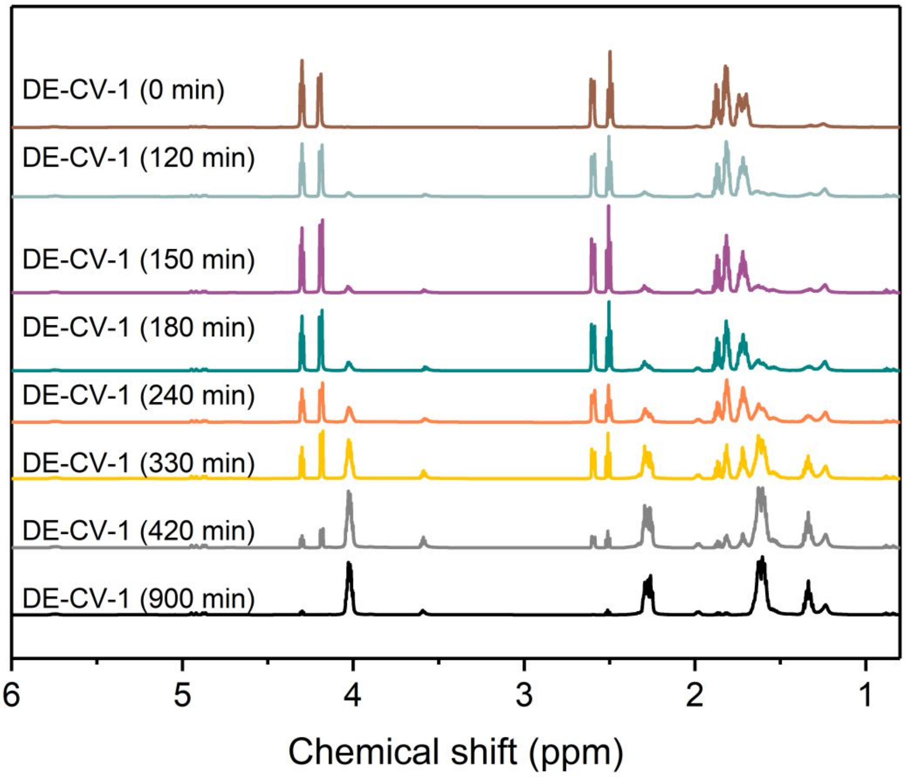

Figure S2-1. ^{1}H-NMR spectra (600 MHz, $CDCl_3$) of the DE-CV-1 during the ring-opening polymerization. The signals from the closed ε-CL and δ-VL rings shift and group after the reaction, which can be used to monitor the conversion as a function of time.

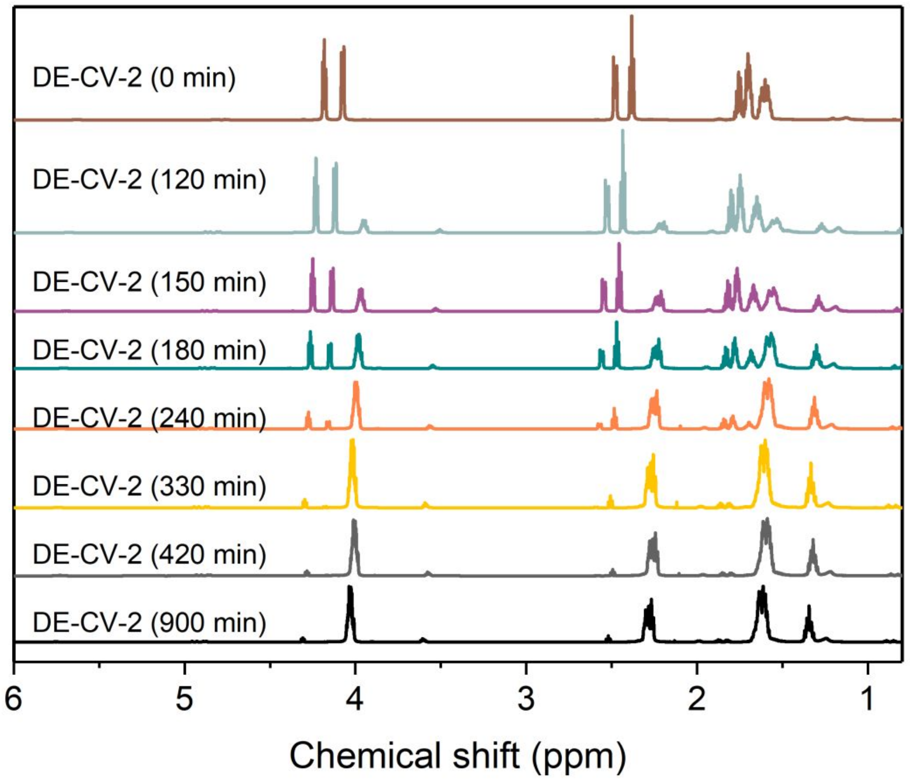

Figure S2-2. ^{1}H-NMR spectra (600 MHz, $CDCl_3$) of the DE-CV-2 during ring-opening polymerization. The signals from the closed ε-CL and δ-VL rings shift and group after the reaction, which can be used to monitor the conversion as a function of time.

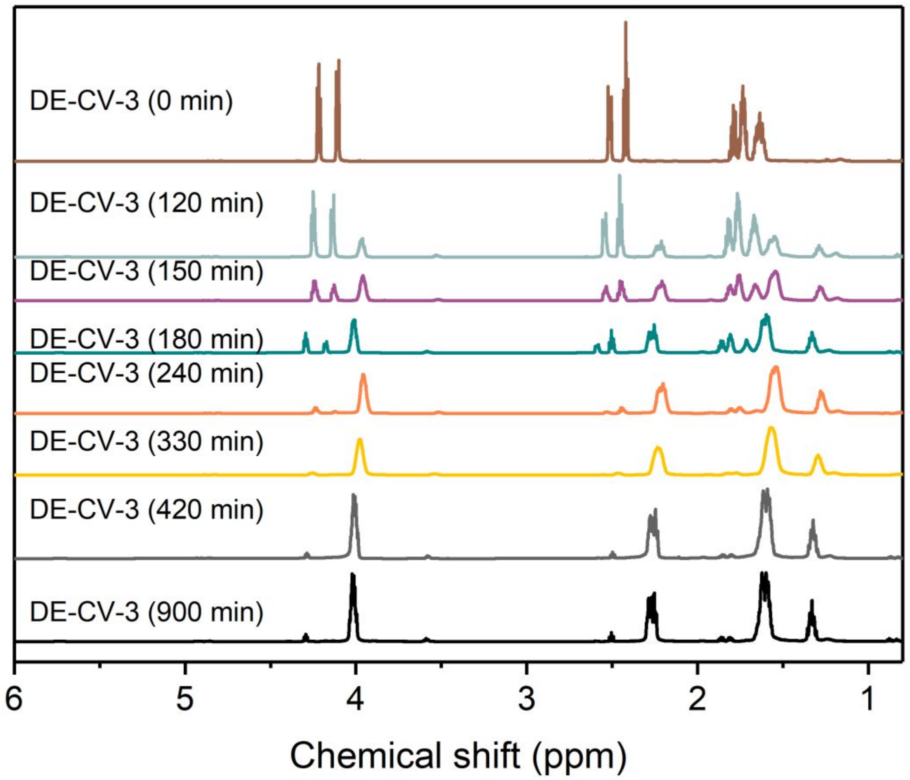

Figure S2-3. ^{1}H-NMR spectra (600 MHz, $CDCl_3$) of the DE-CV-3 during ring-opening polymerization. The signals from the closed ε-CL and δ-VL rings shift and group after the reaction, which can be used to monitor the conversion as a function of time.

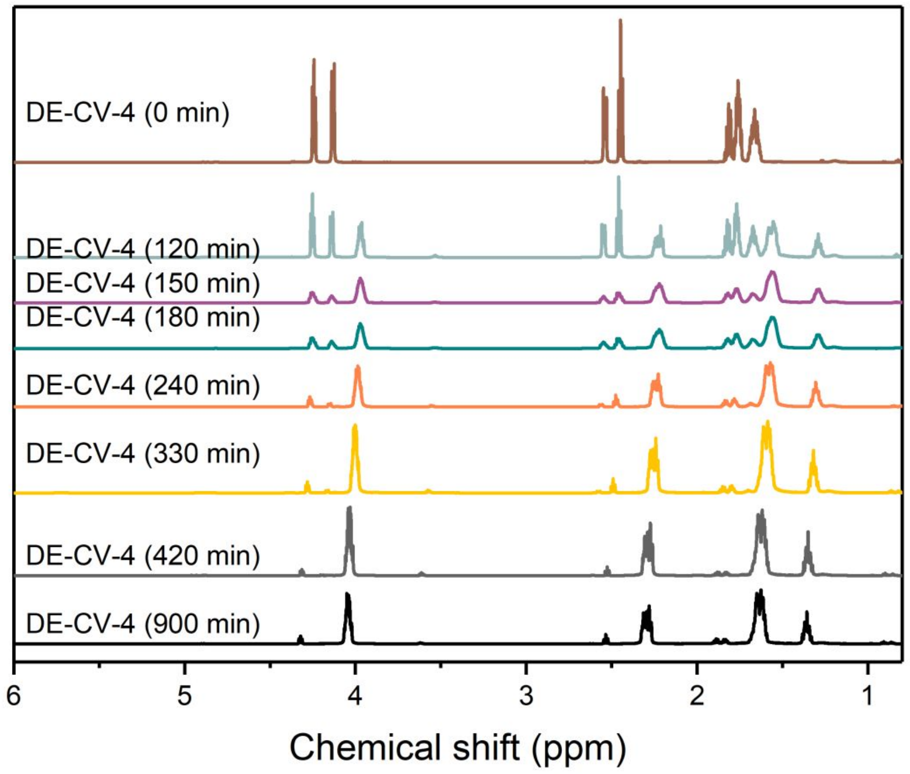

Figure S2-4. ^{1}H-NMR spectra (600 MHz, $CDCl_3$) of the DE-CV-4 during ring-opening polymerization. The signals from the closed ε-CL and δ-VL rings shift and group after the reaction, which can be used to monitor the conversion as a function of time.

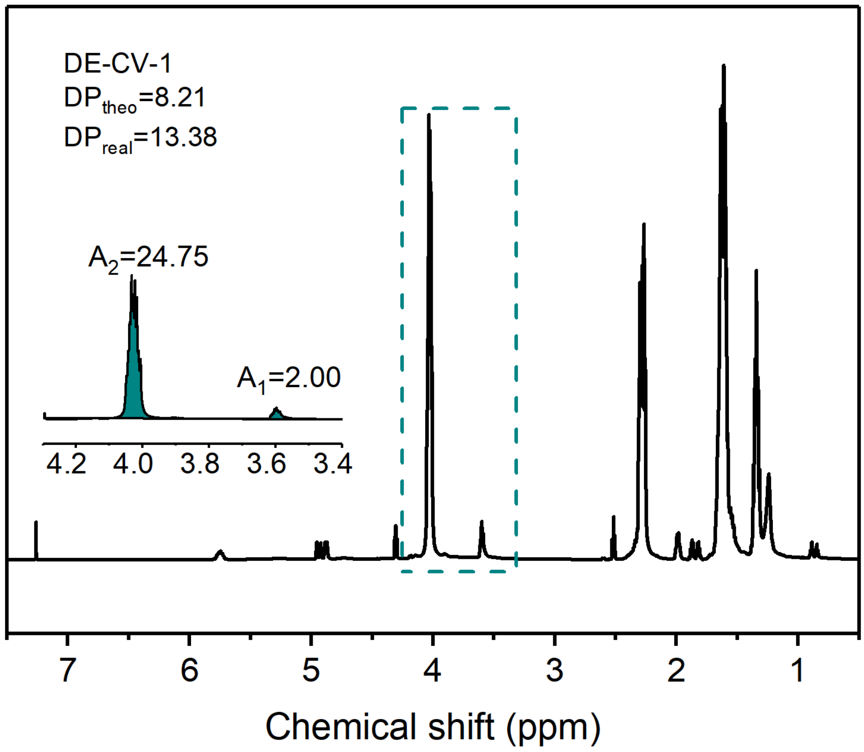

Figure S2-5. ^{1}H-NMR spectrum (600 MHz, $CDCl_3$) of DE-CV-1.

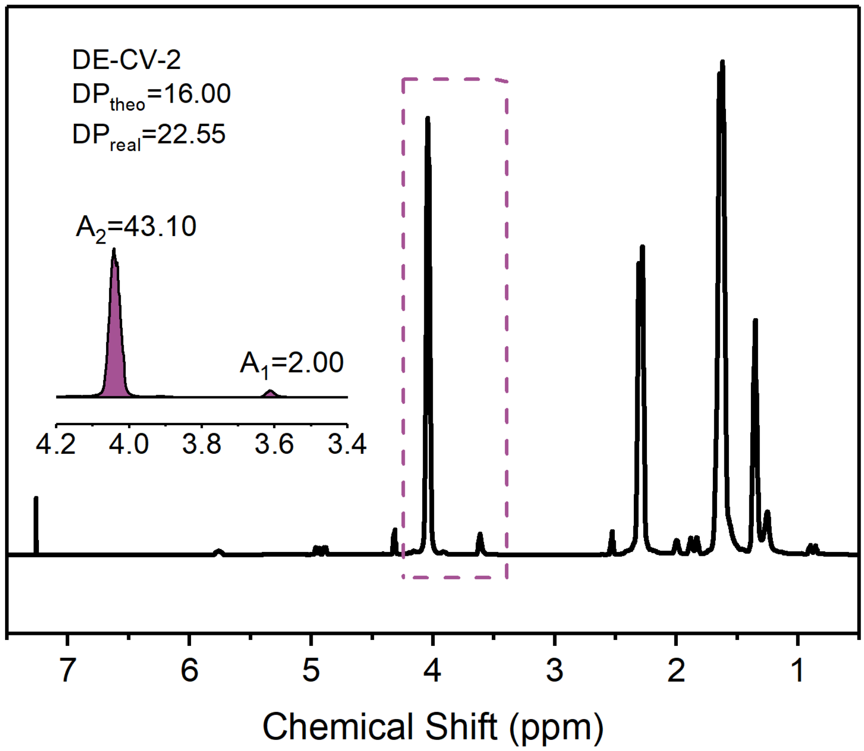

Figure S2-6. ^{1}H-NMR spectrum (600 MHz, $CDCl_3$) of DE-CV-2.

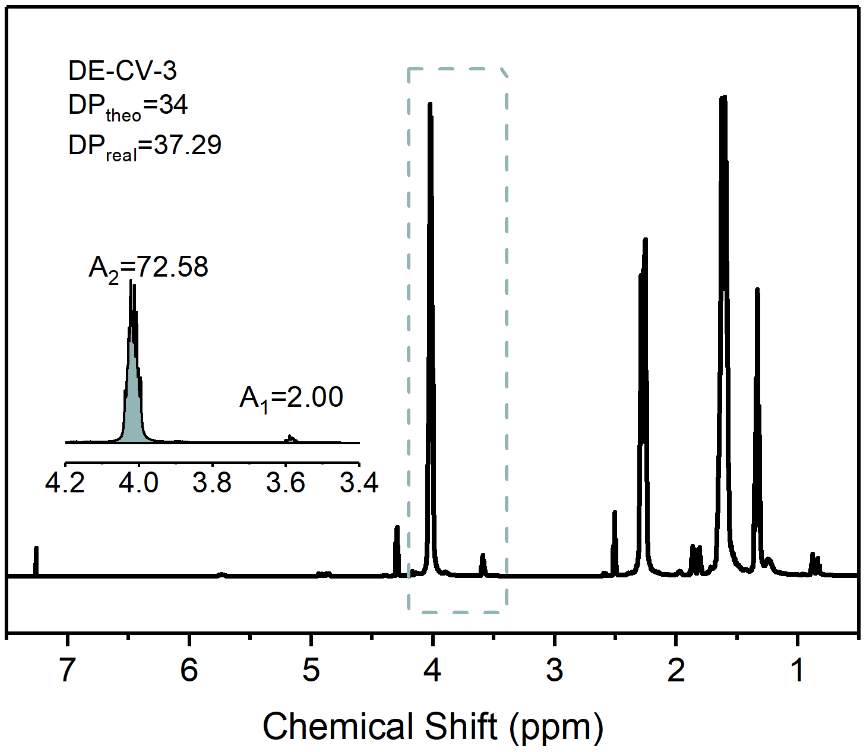

Figure S2-7. ^{1}H-NMR spectrum (600 MHz, $CDCl_3$) of DE-CV-3.

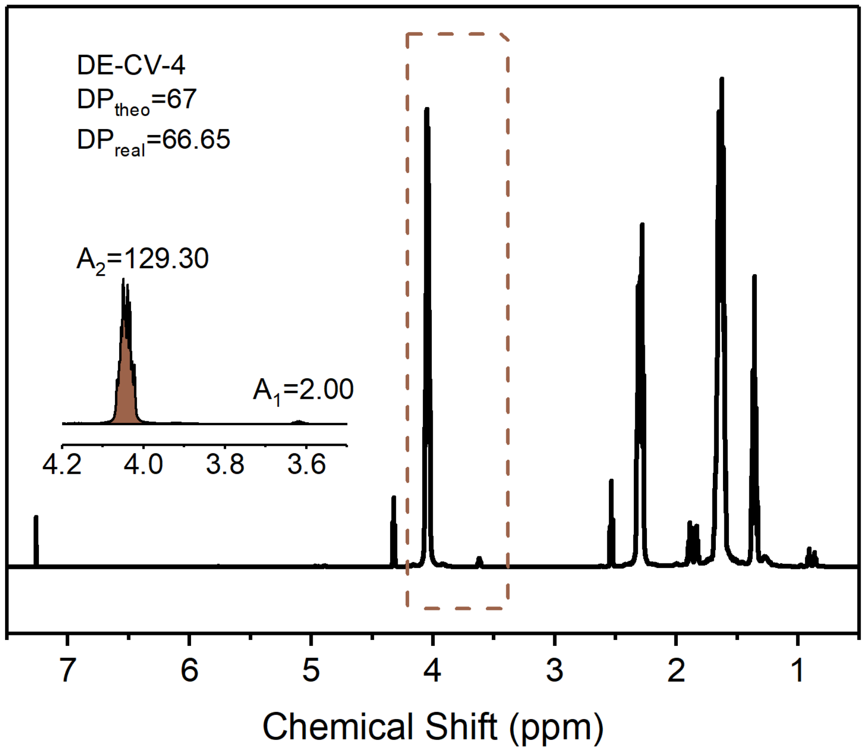

Figure S2-8. ^{1}H-NMR spectrum (600 MHz, $CDCl_3$) of DE-CV-4.

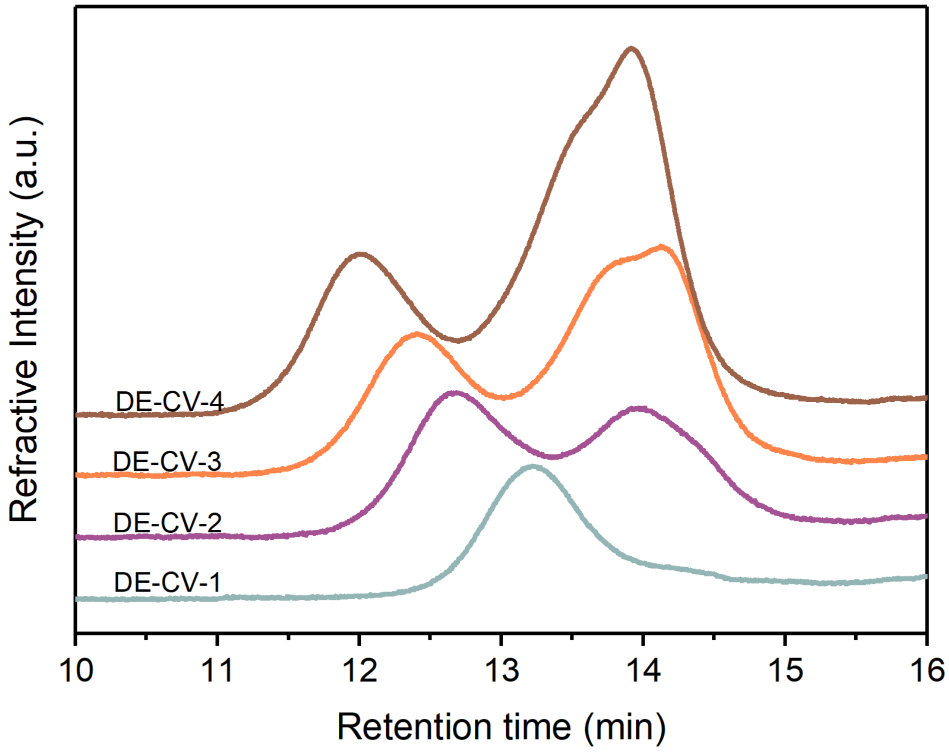

Figure S3. SEC elugrams of the DE-CV-X, measured at 40 ˚C in chloroform.

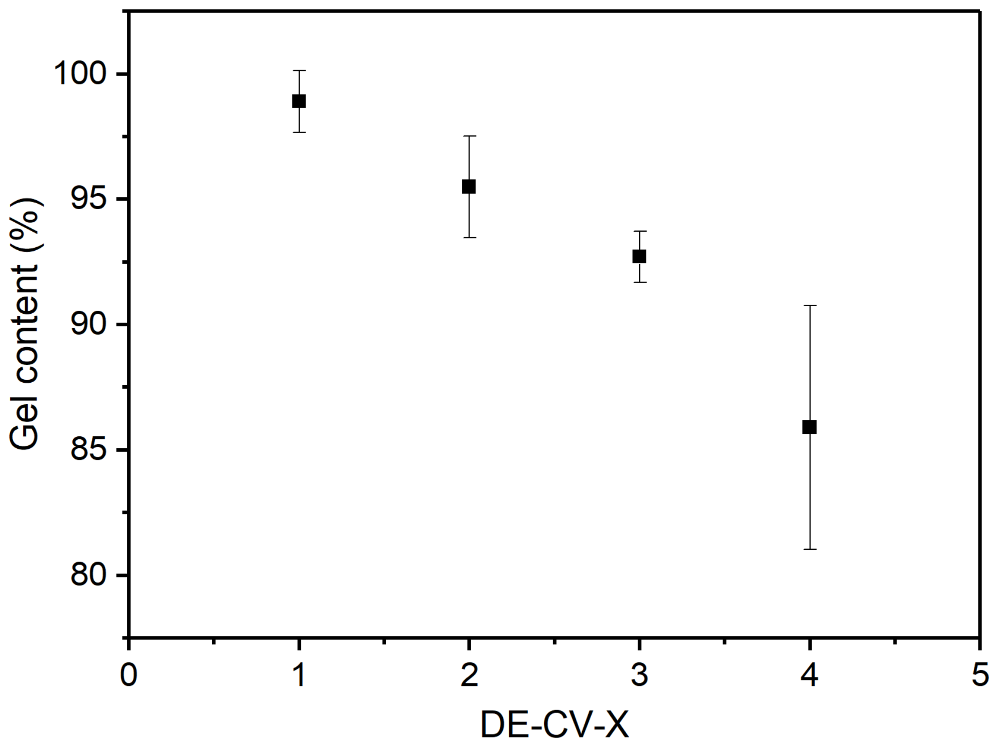

Figure S4. Gel fraction of the ASSETm.

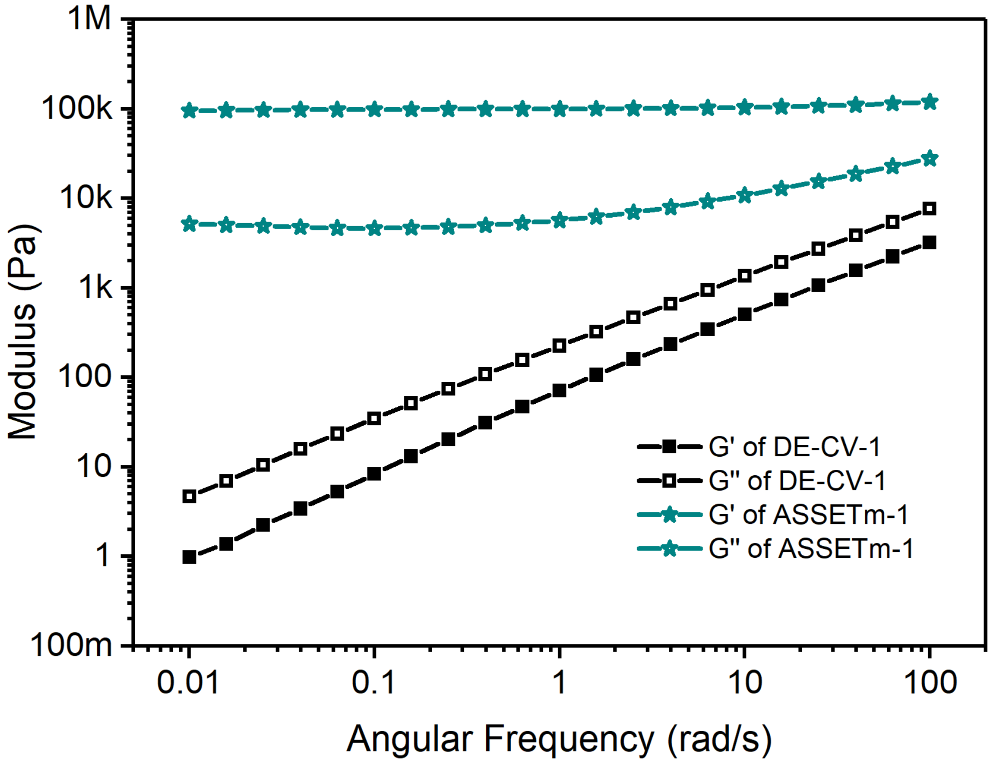

Figure S5-1. Oscillatory rheology measurements on the ASSETm-1.

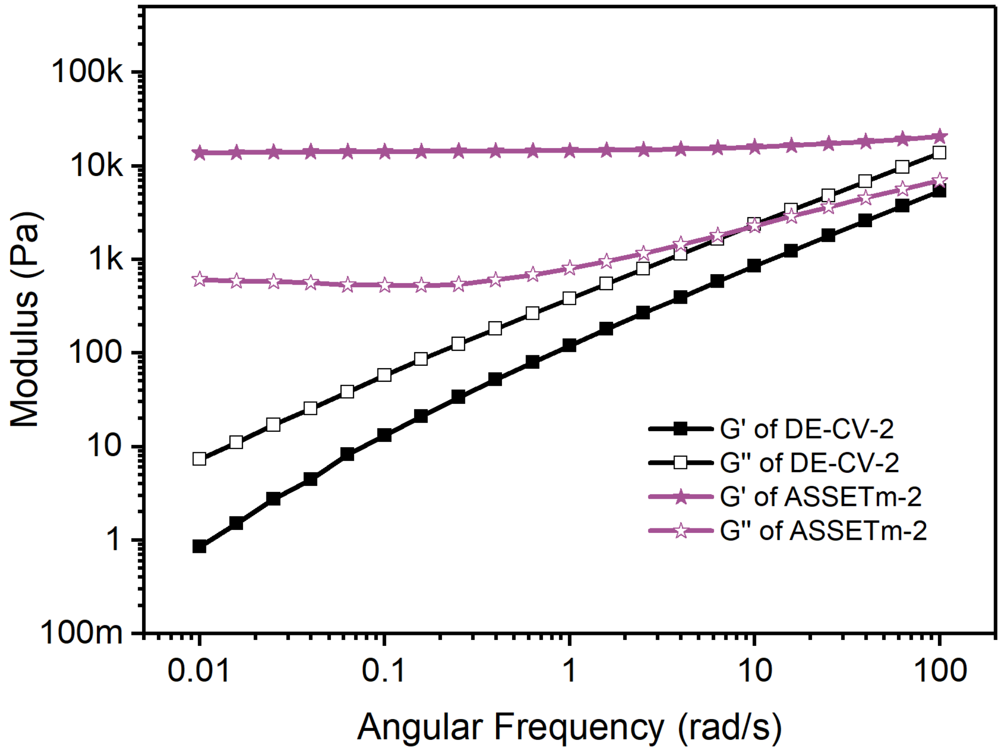

Figure S5-2. Oscillatory rheology measurements on the ASSETm-2.

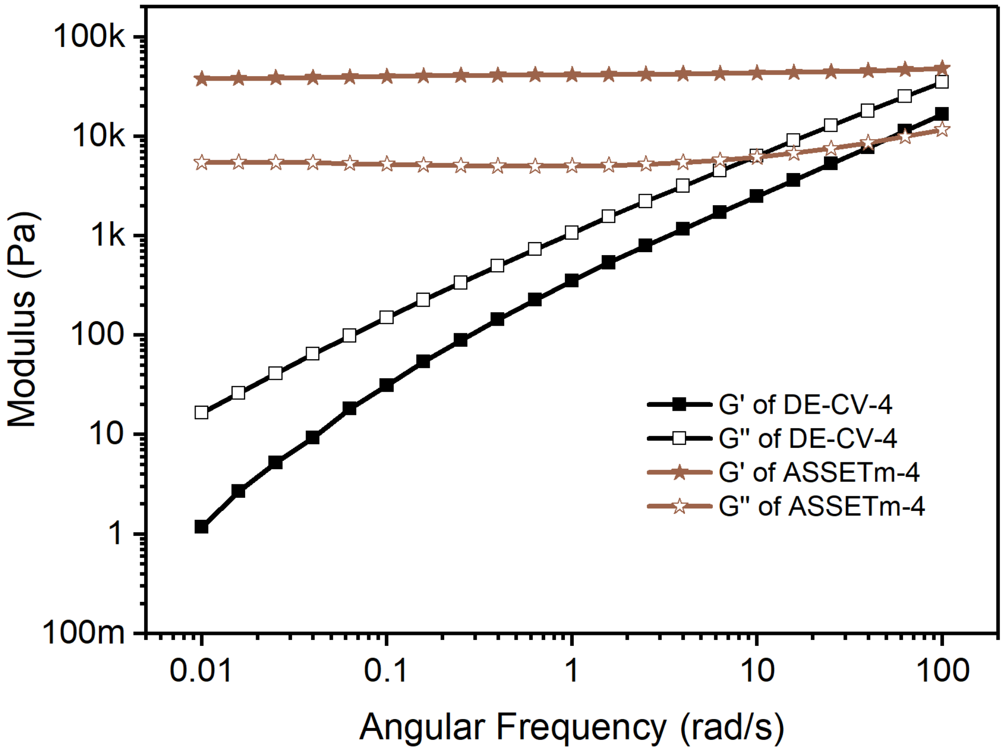

Figure S5-3. Oscillatory rheology measurements on the ASSETm-4.

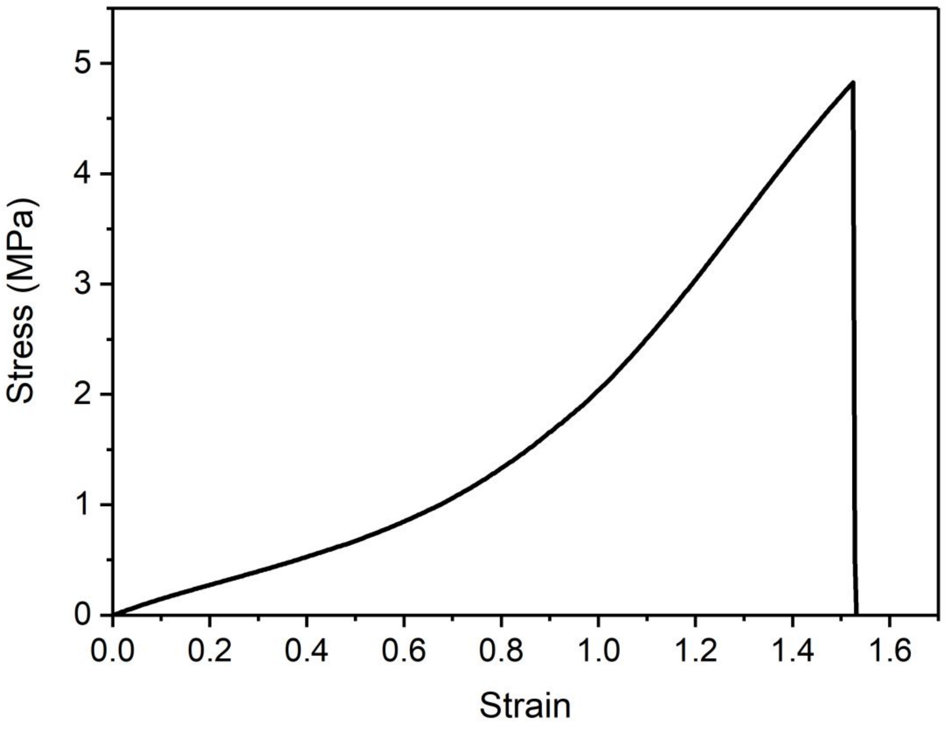

Figure S6. Uniaxial tensile test of the Sylgard 184.

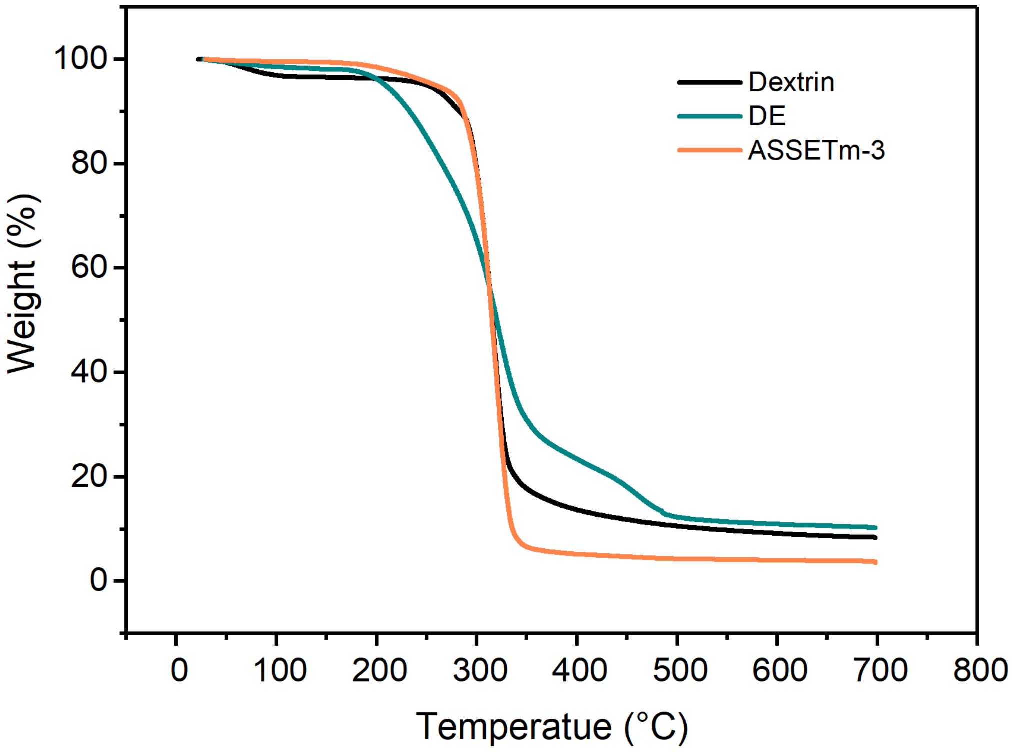

Figure S7-1. Thermogravimetric analyses on the native dextrin, DE, and ASSETm-3.

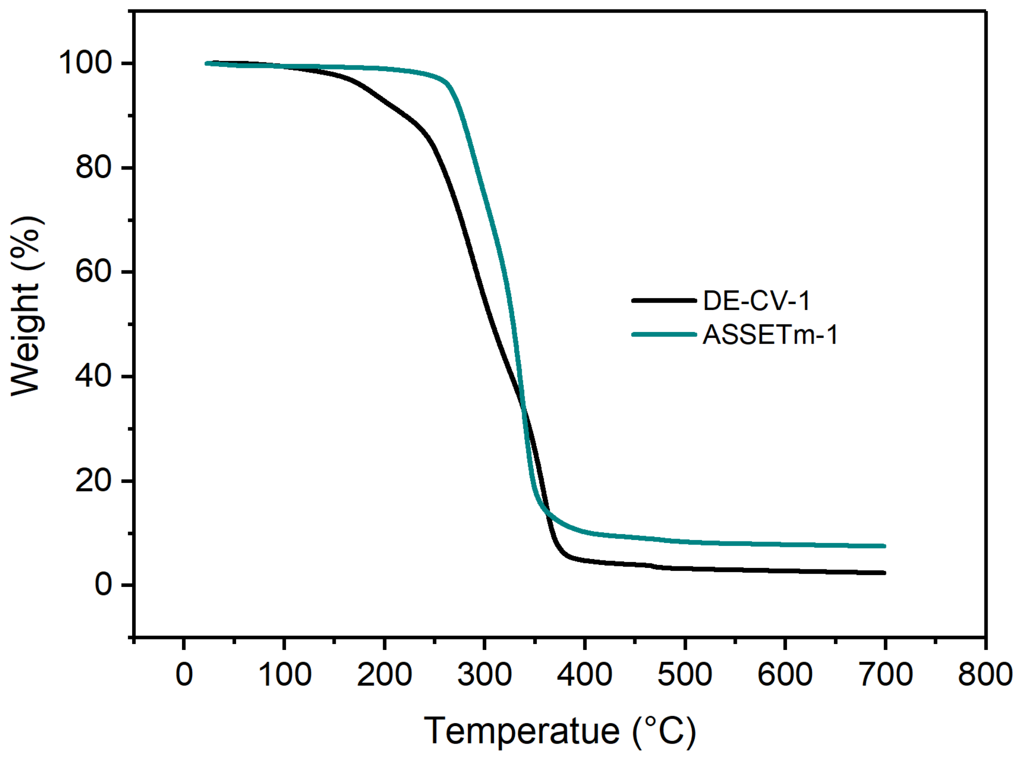

Figure S7-2. Thermogravimetric analyses on the DE-CV-1 and ASSETm-1.

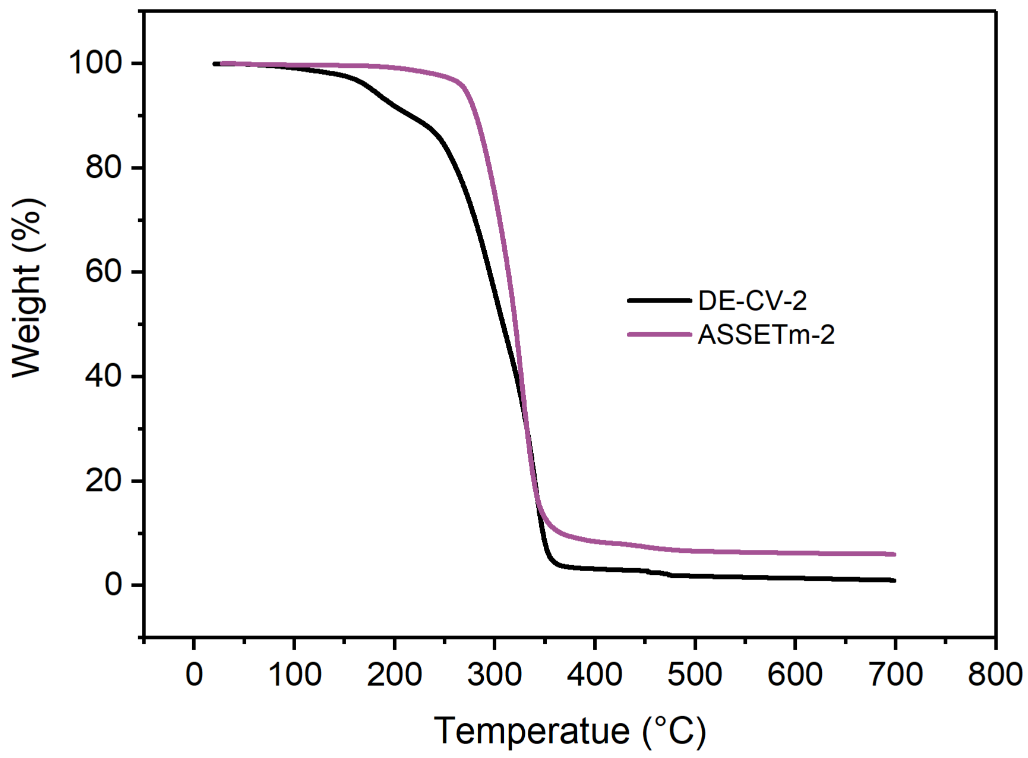

Figure S7-3. Thermogravimetric analyses on the DE-CV-2 and ASSETm-2.

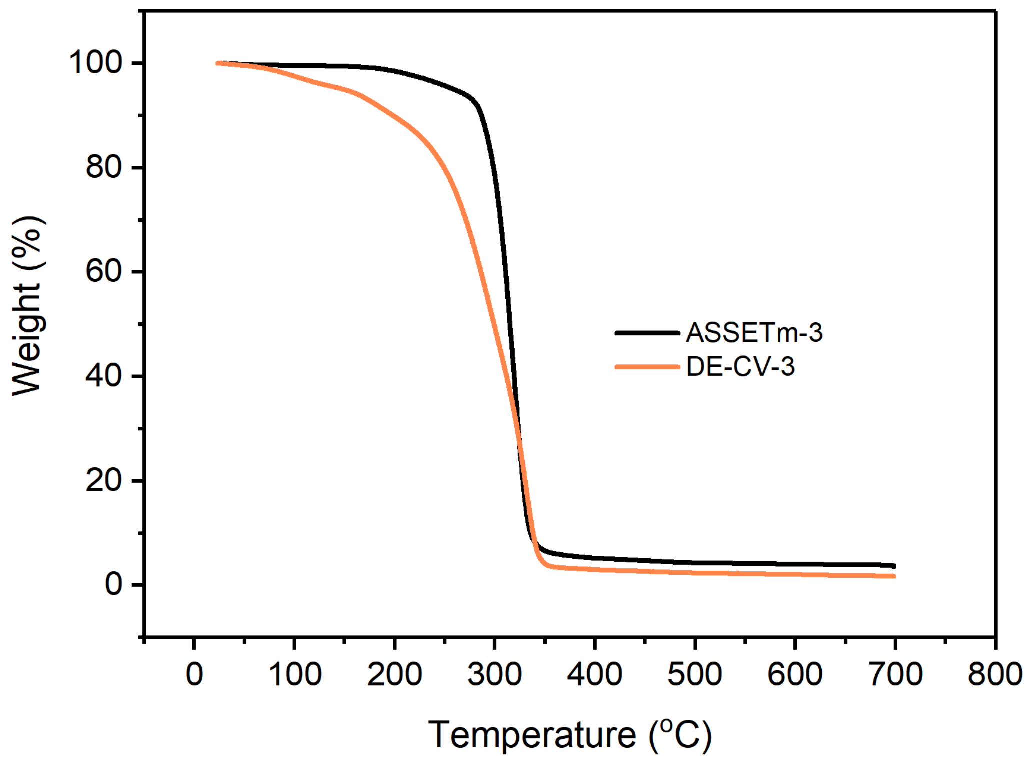

Figure S7-4. Thermogravimetric analyses on the DE-CV-3 and ASSETm-3.

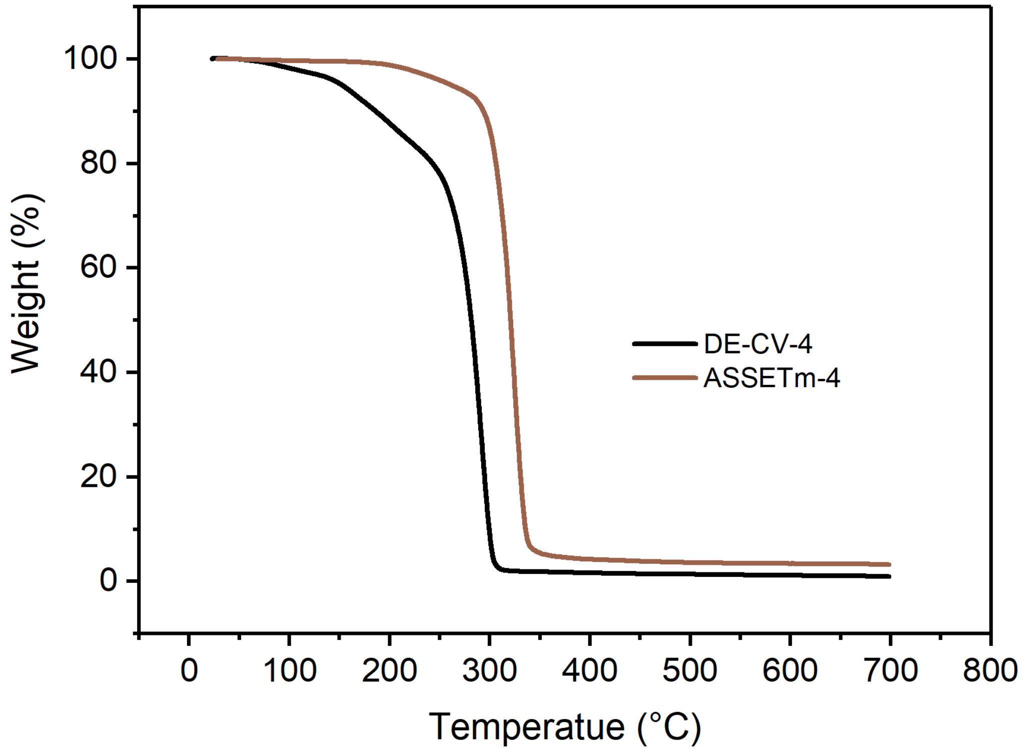

Figure S7-5. Thermogravimetric analyses on the DE-CV-4 and ASSETm-4.

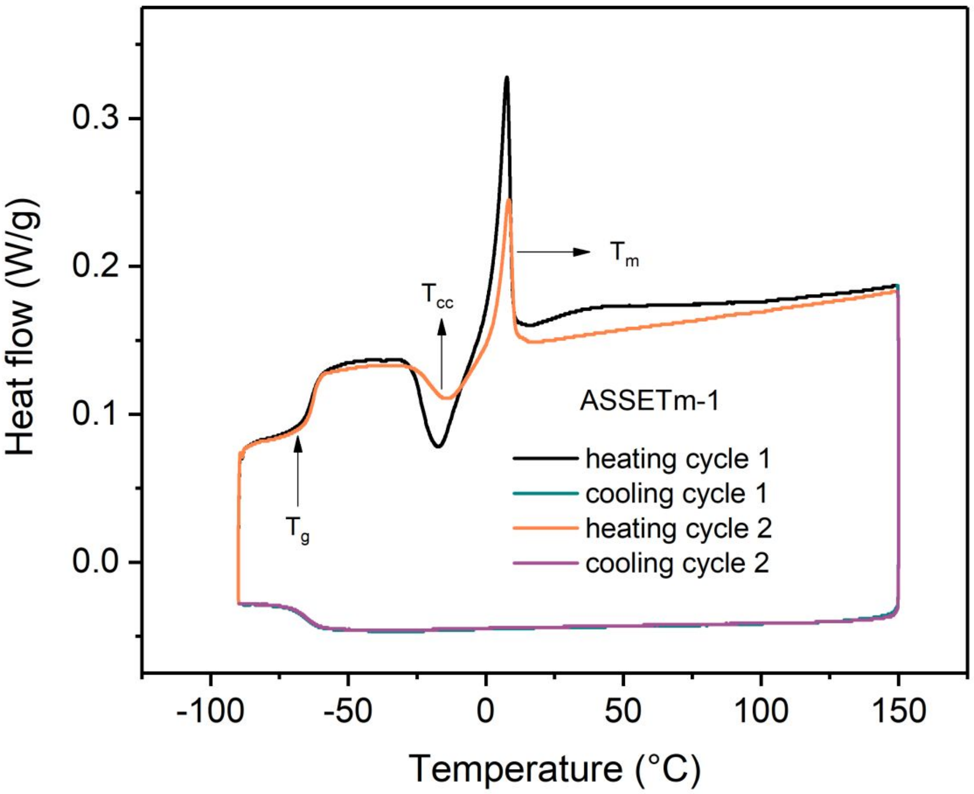

Figure S8-1. Differential scanning calorimetry analyses on the ASSETm-1.

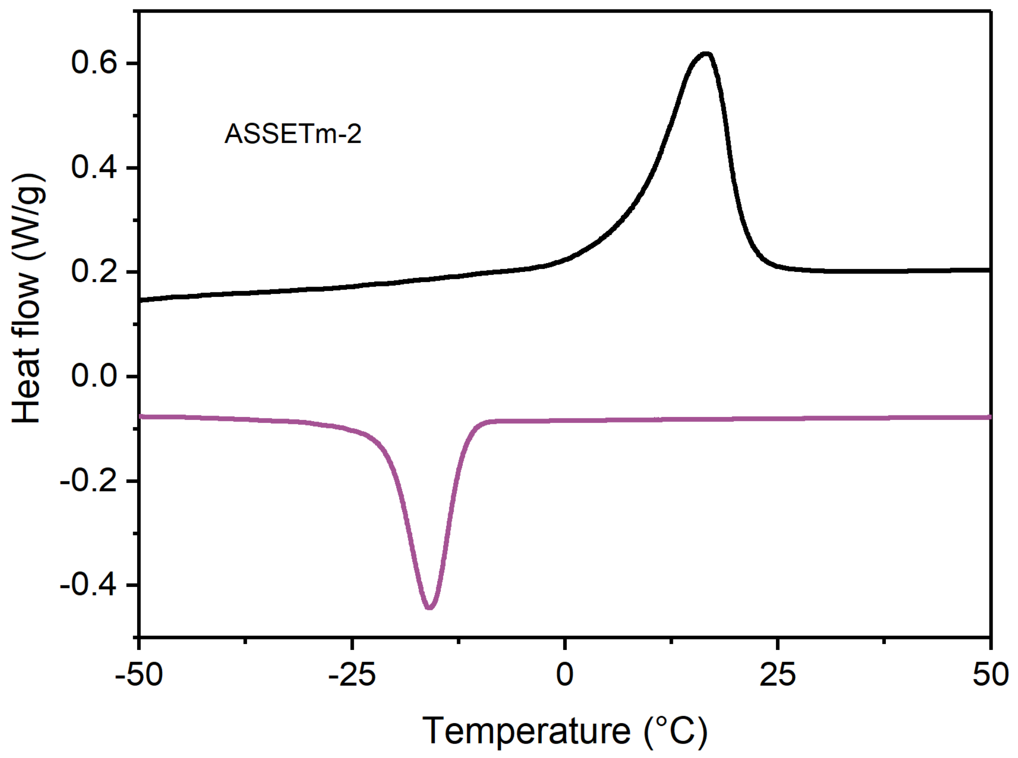

Figure S8-2. Differential scanning calorimetry analyses on the ASSETm-2.

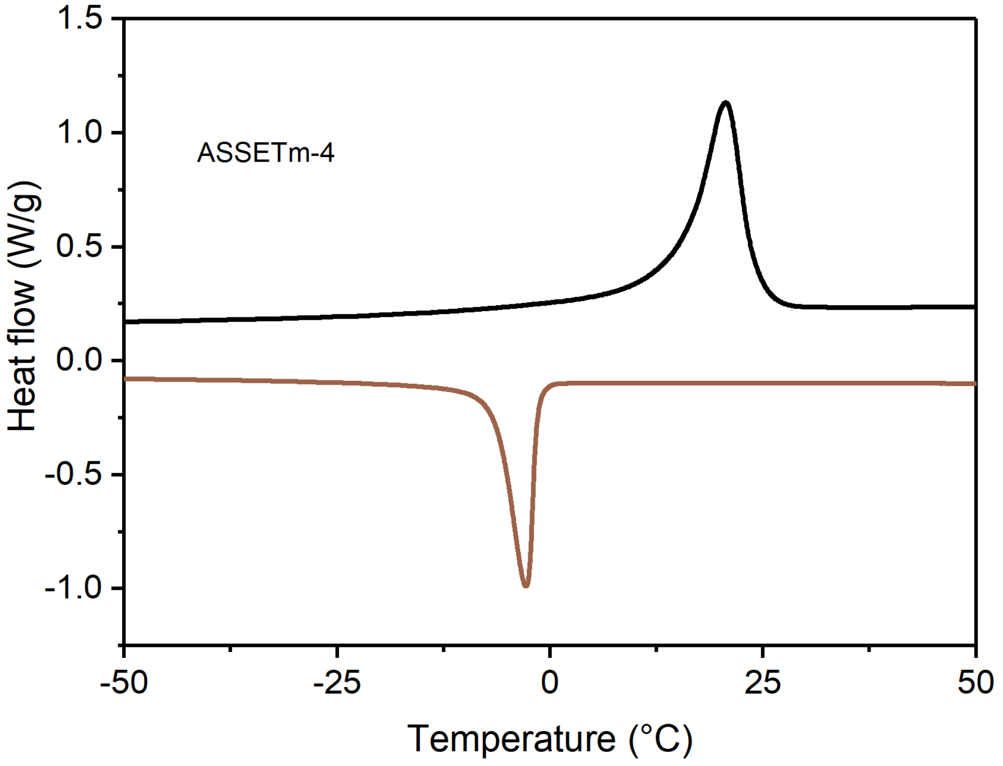

Figure S8-3. Differential scanning calorimetry analyses on the ASSETm-4.

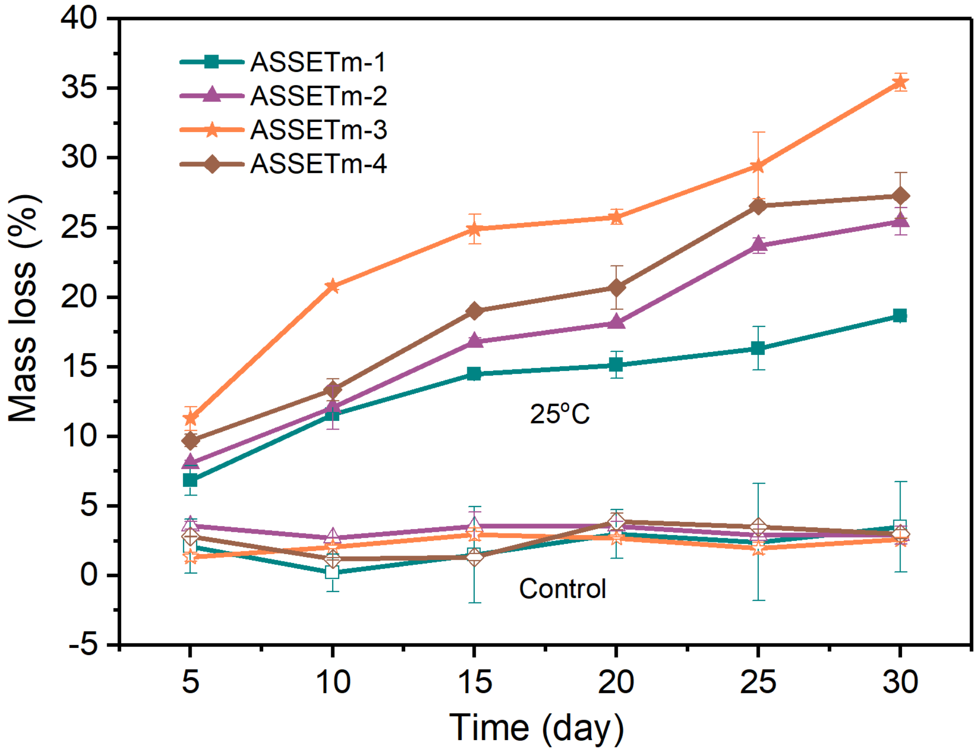

Figure S9. ASSETm's mass loss with enzymatic degradation at 25 ˚C.

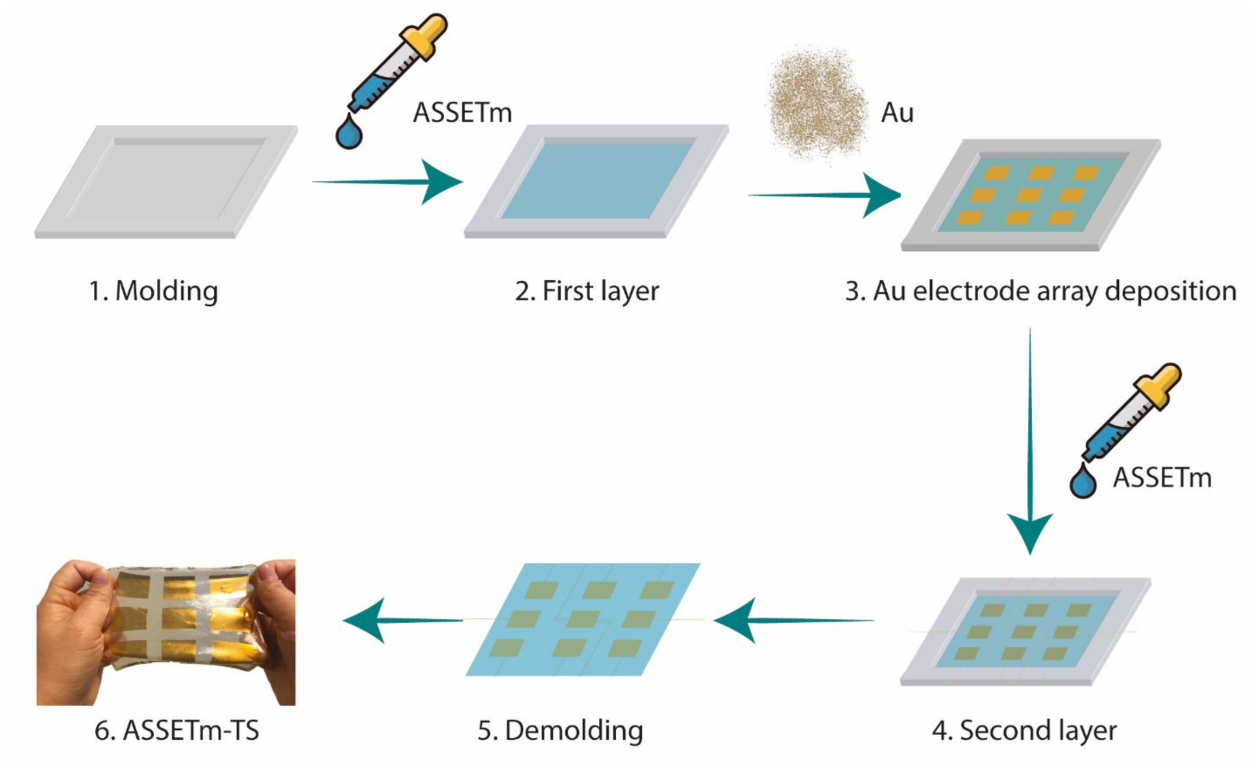

Figure S10. The preparation procedure of the ASSETm-TS.

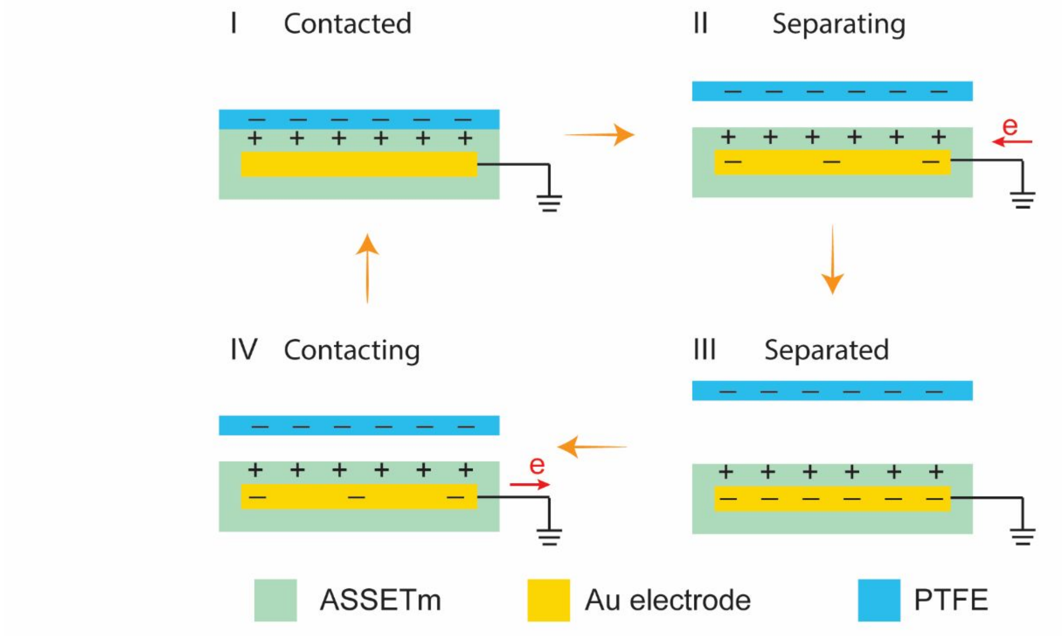

Figure S11. The working mechanism of the ASSETm-TS.

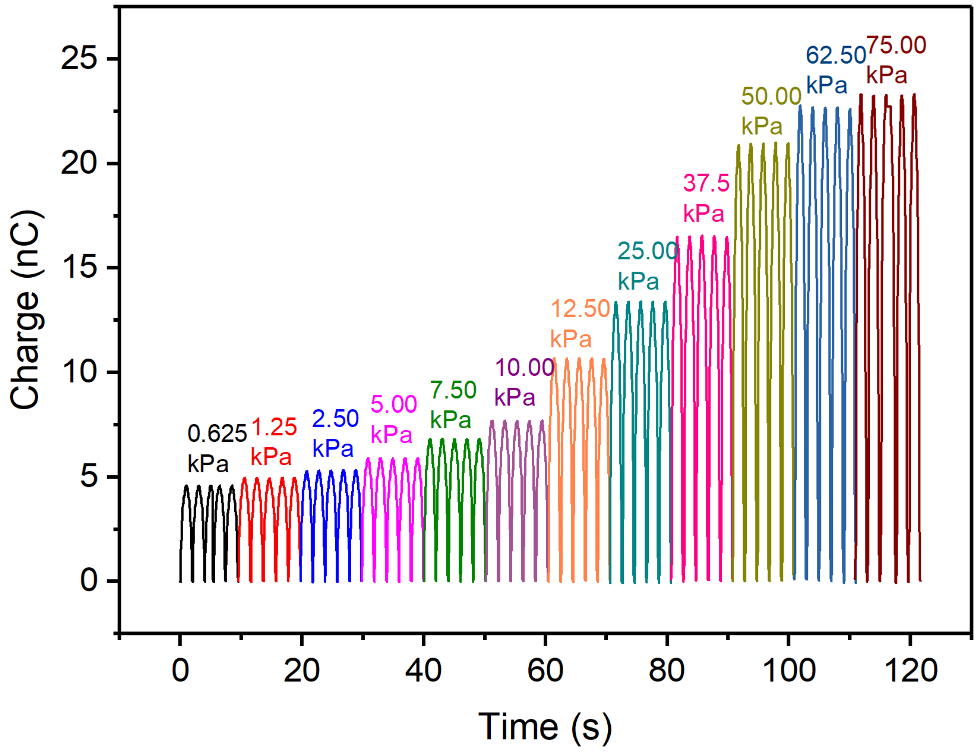

Figure S12-1. Charge changes of the ASSETm-TS at different pressure.

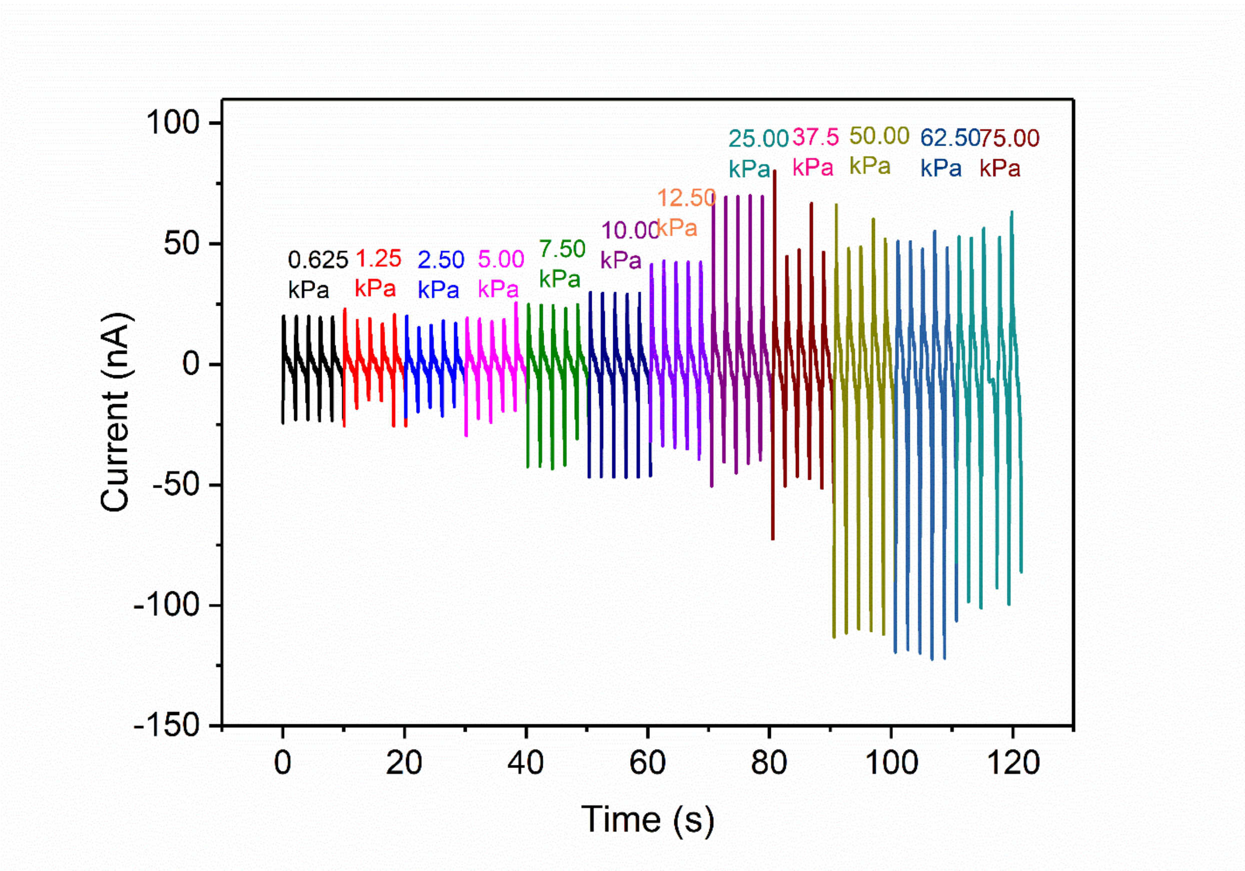

Figure S12-2. Current changes of the ASSETm-TS at different pressure.

Table S1. Composition of the reaction mixtures used for the synthesis of the DE-CV-X

Samples	DE (g)	εCL (g)	δVL (g)	$Sn(Oct)_2$ (g)
DE-CV-1	0.342	1.14	1	0.0405
DE-CV-2	0.342	2.28	2	0.0405
DE-CV-3	0.342	4.56	4	0.0405
DE-CV-4	0.342	9.12	8	0.0405

Table S2. Mechanical properties of the ASSETm

Samples	Young's Modulus (MPa)	Stress at Max. Load (MPa)	Strain at Max Load (%)
ASSETm-1	9.05	0.36	55
ASSETm-2	5.54	0.33	93
ASSETm-3	0.89	0.25	190
ASSETm-4	0.28	0.08	235

Table S3. Thermal characteristics of the dextrin, DE and ASSETm

Samples	T_{deg}‡ (˚C)	T_m± (˚C)	T_c± (˚C)
Dextrin	245	-	-
DE	179	-	-
ASSETm-1	260	6.12	-
ASSETm-2	264	16.64	-16.05
ASSETm-3	275	20.75	-4.70
ASSETm-4	282	20.58	-2.81

‡ temperature corresponding to the maximal decomposition rate observed in TGA using a rate of 10 ˚C min^{-1}, ± determined by differential scanning calorimetry measurements using heating and cooling rates of 10 ˚C min^{-1}.
